# Body mass index and weight change are associated with adult lung function trajectories: the prospective ECRHS study

**DOI:** 10.1136/thoraxjnl-2019-213880

**Published:** 2020-02-25

**Authors:** Gabriela P Peralta, Alessandro Marcon, Anne-Elie Carsin, Michael J Abramson, Simone Accordini, André FS Amaral, Josep M Antó, Gayan Bowatte, Peter Burney, Angelo Corsico, Pascal Demoly, Shyamali Dharmage, Bertil Forsberg, Elaine Fuertes, Vanessa Garcia-Larsen, Thorarinn Gíslason, José-Antonio Gullón, Joachim Heinrich, Mathias Holm, Deborah L Jarvis, Christer Janson, Rain Jogi, Ane Johannessen, Bénédicte Leynaert, Jesús Martínez-Moratalla Rovira, Dennis Nowak, Nicole Probst-Hensch, Chantal Raherison, José-Luis Sánchez-Ramos, Torben Sigsgaard, Valérie Siroux, Giulia Squillacioti, Isabel Urrutia, Joost Weyler, Jan-Paul Zock, Judith Garcia-Aymerich

**Affiliations:** 1 ISGlobal, Barcelona, Spain; 2 Universitat Pompeu Fabra (UPF), Barcelona, Spain; 3 CIBER Epidemiología y Salud Pública (CIBERESP), Barcelona, Spain; 4 Unit of Epidemiology and Medical Statistics, Department of Diagnostics and Public Health, University of Verona, Verona, Italy; 5 IMIM (Hospital del Mar Medical Research Institute), Barcelona, Spain; 6 School of Public Health and Preventive Medicine, Monash University, Melbourne, Victoria, Australia; 7 National Heart and Lung Institute, Imperial College London, London, UK; 8 Allergy and Lung Health Unit, Centre for Epidemiology and Biostatistics, School of Population & Global Health, The University of Melbourne, Melbourne, Victoria, Australia; 9 National Institute of Fundamental Studies, Kandy, Sri Lanka; 10 Division of Respiratory Diseases, IRCCS ‘San Matteo’ Hospital Foundation-University of Pavia, Pavia, Italy; 11 Département de Pneumologie et Addictologie, Hôpital Arnaud de Villeneuve, University Hospital of Montpellier, Montpellier, France; 12 UMr-S 1136 inSerM, iPleSP, UPMc, Sorbonne Universités, Paris, France; 13 Section of Sustainable Health, Department of Public Health and Clinical Medicine, Umeå University, Umeå, Sweden; 14 Program in Human Nutrition, Department of International Health, Johns Hopkins Bloomberg School of Public Health, Baltimore, Maryland, USA; 15 Department of Sleep, Landspitali University Hospital Reykjavik, Reykjavik, Iceland; 16 Medical Faculty University of Iceland, Reykjavik, Iceland; 17 Department of Pneumology, Hospital San Agustin, Avilés, Spain; 18 Institute and Outpatient Clinic for Occupational, Social and Environmental Medicine, University Hospital Munich (LMU), Munich, Germany; 19 Comprehensive Pneumology Center Munich (CPC-M), German Center for Lung Research (DZL), Munich, Germany; 20 Department of Occupational and Environmental Medicine, Sahlgrenska University Hospital, Gothenburg, Sweden; 21 MRC-PHE Centre for Environment and Health, Imperial College London, London, UK; 22 Department of Medical Sciences, Respiratory, Allergy and Sleep Research, Uppsala University, Uppsala, Sweden; 23 Lung Clinic, Tartu University Hospital, Tartu, Estonia; 24 Department of Global Public Health and Primary Care, University of Bergen, Bergen, Norway; 25 Department of Occupational Medicine, Haukeland University Hospital, Bergen, Norway; 26 INSERM U1168, VIMA (Aging and Chronic Diseases. Epidemiological and Public Health Approaches), Villejuif, France; 27 UMR-S 1168, Univ Versailles St-Quentin-en-Yvelines, St-Quentin-en-Yvelines, France; 28 Facultad de Medicina de Albacete, Universidad de Castilla - La Mancha, Albacete, Spain; 29 Swiss Tropical and Public Health Institute, Basel, Switzerland; 30 Department of Public Health, University of Basel, Basel, Switzerland; 31 INSERM U897, Institute of Public Health and Epidemiology, Bordeaux University, Bordeaux, France; 32 Department of Nursing, University of Huelva, Huelva, Spain; 33 Department of Public Health, Section for Environment Occupation and Health, Danish Ramazzini Centre, Aarhus University, Aarhus, Denmark; 34 Institute for Advanced Biosciences, UGA-Inserm U1209-CNRS UMR 5309, Team of Environmental Epidemiology Applied to Reproduction and Respiratory Health, Grenoble, France; 35 Department of Public Health and Pediatrics, University of Turin, Turin, Italy; 36 Department of Respiratory, Galdakao Hospital, Galdakao, Spain; 37 Department of Epidemiology and Social Medicine, University of Antwerp, Antwerp, Belgium

**Keywords:** adults, BMI, lung function, obesity, weight change, epidemiology

## Abstract

**Background:**

Previous studies have reported an association between weight increase and excess lung function decline in young adults followed for short periods. We aimed to estimate lung function trajectories during adulthood from 20-year weight change profiles using data from the population-based European Community Respiratory Health Survey (ECRHS).

**Methods:**

We included 3673 participants recruited at age 20–44 years with repeated measurements of weight and lung function (forced vital capacity (FVC), forced expiratory volume in 1 s (FEV_1_)) in three study waves (1991–93, 1999–2003, 2010–14) until they were 39–67 years of age. We classified subjects into weight change profiles according to baseline body mass index (BMI) categories and weight change over 20 years. We estimated trajectories of lung function over time as a function of weight change profiles using population-averaged generalised estimating equations.

**Results:**

In individuals with normal BMI, overweight and obesity at baseline, moderate (0.25–1 kg/year) and high weight gain (>1 kg/year) during follow-up were associated with accelerated FVC and FEV_1_ declines. Compared with participants with baseline normal BMI and stable weight (±0.25 kg/year), obese individuals with high weight gain during follow-up had −1011 mL (95% CI −1.259 to −763) lower estimated FVC at 65 years despite similar estimated FVC levels at 25 years. Obese individuals at baseline who lost weight (<−0.25 kg/year) exhibited an attenuation of FVC and FEV_1_ declines. We found no association between weight change profiles and FEV_1_/FVC decline.

**Conclusion:**

Moderate and high weight gain over 20 years was associated with accelerated lung function decline, while weight loss was related to its attenuation. Control of weight gain is important for maintaining good lung function in adult life.

**Key questions**

**What is the key question?**

- Is weight change over a 20-year period associated with lung function trajectories in adult life?

**What is the bottom line?**

- Moderate and high weight gain over a 20-year period was associated with accelerated FVC and FEV_1_ decline, while weight loss was related to its attenuation.

**Why read on?**

- This study, which is based on data collected as part of the multicentre prospective ECRHS study, reinforces the public health message that overweight and obesity have deleterious effects on respiratory health. However, these negative effects can be reversed by weight loss even later in adult life.

## Background

Lung function is a significant predictor of future morbidity and mortality in the general population.^1^ Maintaining good lung function across adult life is important to prevent chronic respiratory diseases, which nowadays represent a serious public health problem around the world.^2^ There is consistent evidence showing that overweight, obesity and weight gain in adulthood are detrimental to lung function, as described by the forced vital capacity (FVC) and/or forced expiratory volume in 1 s (FEV_1_). Previous population-based and occupational cohort studies have shown that excessive weight gain in adulthood is associated with lower lung function levels and with an increased rate of lung function decline independently of age and smoking status.^3–8^ Another longitudinal study in healthy young adults (age range at baseline 18–30 years) showed that lung function was lower both with higher baseline body mass index (BMI) and with increasing BMI over a 10-year period.^9^ Similarly, a population-based study of young adults (mean age at baseline 41 years) analysing the effects of changes in obesity status on lung function found that remaining or becoming obese accelerated lung function decline over an 8-year follow-up, while becoming non-obese was related to its attenuation.^10^

All these previous studies have had relatively short follow-up periods (up to 10 years) and most investigated this link only up to 50 years of age. This precludes a more comprehensive understanding of the role of weight change on lung function during adulthood and older life and supports the need for further studies with longer follow-up periods extending into late adult life. Understanding the effects of weight changes on lung function during adult life is of utmost importance given the epidemic levels of overweight and obesity globally.^11^

The European Community Respiratory Health Survey (ECRHS) is a large multicentre population-based study with available measures of weight, height and lung function at three time points over a 20-year period, as well as detailed information of sociodemographic and lifestyle factors from adults living across Europe and Australia.^12–14^ Under the framework of the Ageing Lungs in European Cohorts (ALEC) consortium (www.alecstudy.org), we aimed to assess the lung function trajectories of adults of the ECRHS study according to different weight change profiles that combined BMI at baseline and weight change over a 20-year period.

## Methods

### Study population

The ECRHS started in 1991–1993 (ECRHS I), when over 18 000 young adults aged 20–44 years were randomly recruited from available population-based registers (population-based arm), with an oversampling of asthmatics (symptomatic arm). Participants were followed up in 1999–2003 (ECRHS II) and 2010–2014 (ECRHS III) when they were aged 27–57 and 39–67 years, respectively. More details of the study design are available elsewhere.^12–14^ In this analysis we included participants who had weight at ECRHS I and III and lung function and base covariates (sex, age, height and smoking status) at all three surveys (3673 participants from 26 centres in 12 countries) (see online supplementary figure S1).

10.1136/thoraxjnl-2019-213880.supp1 Supplementary data

Ethical approval was obtained from the ethics committees of all participating institutions and all participants provided informed written consent.

### Lung function

Lung function was measured by spirometry at ECRHS I, II and III. Centres used different spirometers at ECRHS I and II, but almost all centres used the same spirometer at ECRHS III (see online supplementary table S1). In the three examinations, forced vital capacity (FVC) and forced expiratory volume in 1 s (FEV_1_), repeatable to 150 mL from at least two of a maximum of five correct manoeuvres that met the American Thoracic Society and European Respiratory Society recommendations,^15^ were used as the primary outcomes. The FEV_1_/FVC ratio was also analysed. In the present analysis, we used lung function measurements collected pre-bronchodilator. We also calculated lung function SD scores (z-scores) using the Global Lung Initiative (GLI) equation references,^16^ and used these variables as secondary outcomes.

### Weight change profiles

BMI was calculated by dividing measured weight (kg) by measured height (m) squared. We defined categories of BMI at ECRHS I (baseline) as ‘underweight’ (BMI <20 kg/m^2^), ‘normal weight’ (20 kg/m^2^≤BMI<25 kg/m^2^), ‘overweight’ (25 kg/m^2^≤BMI <30 kg/m^2^) and ‘obese’ (BMI ≥30 kg/m^2^), as in previous ECRHS studies.^8^ We computed weight change during follow-up as the difference between weight measured at ECRHS III and ECRHS I divided by the total time of follow-up (in years) and categorised it into stable weight, weight loss and weight gain. Since there are no standard references for weight change in adults, we used similar weight change categories as in a recent longitudinal long-term population-based study^17^: ‘weight loss’ (<−0.25 kg/year), ‘stable weight' (±0.25 kg/year), ‘moderate weight gain’ (>0.25 to ≤1 kg/year) and ‘high weight gain’ (>1 kg/year). We combined baseline BMI categories with weight change categories to classify participants in weight change profiles. This combined variable was used as the main exposure variable in the analysis.

### Other relevant variables

Sociodemographic and other health data were collected using questionnaires. These included sex, age, age completed full-time education (<17 years; 17–20 years;>20 years), smoking status (never smoker; ex-smoker; current smoker), secondhand smoke exposure (yes; no) and asthma (yes; no). Current asthma was defined as having reported physician-diagnosed asthma and at least one of the following: asthma-like symptoms (wheeze, nocturnal chest tightness, attacks of breathlessness after activity/at rest/at night-time), asthma attacks, use of inhaled/oral medicines for breathing problems (in the last 12 months), or current use of inhalers, aerosols or tablets for asthma. Leisure-time vigorous physical activity was assessed at ECRHS II by asking participants how often and for how many hours per week they usually exercised so much that they got out of breath or sweaty. Participants were categorised as being active if they exercised with a frequency of two or more times a week and with a duration of about 1 hour a week or more, and non-active otherwise.^18^ Finally, at ECRHS II participants reported if they presented any of the following long-term limiting illnesses: hypertension, heart disease, diabetes, cancer or stroke.

### Statistical analysis

We used population-averaged generalised estimating equations (GEE) to estimate lung function trajectories from age 20 to 67 years (the full age range of the study sample) as a function of weight change profiles. Prior to stratifying models by weight change profiles, we tested the interaction between age, BMI at baseline and weight change, and we found that it was statistically significant for all lung function parameters (p value <0.01 for all models). All GEE models had the individuals as the clustering factor (to account for repeated lung function measurements at ECRHS I, II and III) and an unstructured within-cluster correlation. GEE models had FVC, FEV_1_ or FEV_1_/FVC as the outcome variables. Interaction terms between age (or age squared) and weight change profiles were entered to allow for different trajectories of lung function with ageing across weight change profiles. We entered sex as a fixed covariate and height, age, age squared, smoking status, current asthma and spirometer type as time-specific covariates. We also included an interaction term between smoking status and age (to account for a faster decline over time in smokers). We centred continuous variables at the mean (over the data from the three examinations) before modelling. Adjusted lung function over age was calculated by setting continuous and categorical variables equal to the mean and proportion, respectively (calculated over the study sample).

In a secondary analysis we repeated the models using lung function z-scores instead of absolute lung function values. To assess whether estimated lung function trajectories differed by sex we tested for sex interactions (by including an interaction term between sex and weight change profiles) and we stratified final models by sex. We performed several sensitivity analyses to assess the robustness of the estimated lung function trajectories to various assumptions regarding confounding, change of spirometry devices or weight change categorisation (see online supplementary file).

All analyses were conducted following a complete case approach in Stata/SE 14.0 (StataCorp, College Station, Texas, USA).

## Results

### Characteristics of the study sample

Compared with those not included in the present analysis (n=12 909), individuals who were included were slightly older, less likely to be current smokers, be exposed to secondhand smoke and had higher educational levels at ECRHS I, but they did not differ in terms of weight, BMI and lung function (see online supplementary table S2). Table 1 shows the main characteristics of the study sample (n=3673). Mean (SD) age of the study sample was 34.3 (7.1) years at baseline and 54.3 (7.1) years at the last follow-up. Approximately half of the study sample were women (53.3%) and 40% had completed full-time education when they were 20 years of age or older.

**Table 1:** Characteristics of the study sample*

Characteristics	ECRHS I	ECRHS II	ECRHS III
	N (%) or mean (SD)	N (%) or mean (SD)	N (%) or mean (SD)
Symptomatic study arm	544 (14.8)	–	–
Women	1956 (53.3)	–	–
Age in years	34.3 (7.1)	43.0 (7.0)	54.3 (7.1)
Height in cm	170.6 (9.4)	170.3 (9.4)	169.4 (9.5)
Weight in kg	69.5 (13.5)	74.0 (15.1)	77.9 (16.1)
BMI			
Continuous, in kg/m^2^	23.8 (3.7)	25.4 (4.3)	27.1 (4.9)
Underweight	453 (12.3)	222 (6.1)	119 (3.2)
Normal weight	2097 (57.1)	1676 (45.8)	1224 (33.3)
Overweight	892 (24.3)	1298 (35.5)	1481 (40.3)
Obese	231 (6.3)	461 (12.6)	849 (23.1)
Smoking status			
Non-smoker	1651 (45.0)	1576 (42.9)	1518 (41.3)
Ex-smoker	818 (22.3)	1119 (30.5)	1500 (40.8)
Current smoker	1204 (32.8)	978 (26.6)	655 (17.8)
Secondhand smoke exposure, yes	1939 (52.9)	1321 (36.1)	680 (18.6)
Current asthma, yes†	378 (10.5)	491 (13.8)	570 (16.2)
Age completed full-time education			
<17 years	675 (21.5)	–	–
17–20 years	1205 (38.4)	–	–
>20 years	1256 (40.1)	–	–
Physical activity. Active status‡	–	1363 (52.2)	–
Any long-term limiting illness, yes§	–	405 (17.1)	–
Lung function			
FVC (mL)	4516 (988)	4354 (980)	3964 (948)
FEV_1_ (mL)	3702 (798)	3485 (790)	3006 (753)
FEV_1_/FVC (%)	82.3 (6.9)	80.3 (6.5)	75.8 (6.5)
Lung function (z-scores)¶			
FVC z-score	0.01 (0.95)	0.02 (1.00)	−0.08 (0.94)
FEV_1_ z-score	−0.01 (1.06)	−0.03 (1.08)	−0.34 (1.04)
FEV_1_/FVC z-score	−0.06 (1.03)	−0.10 (1.00)	−0.48 (0.89)

*Some variables had missing values. Number of missing values for ECRHS I: 10 in secondhand smoke exposure, 78 in current asthma, and 537 in age completed full-time education. Number of missing values for ECRHS II: 18 in secondhand smoke exposure, 118 in current asthma, 1062 in physical activity and 1300 in any long-term limiting illness. Number of missing values for ECRHS III: 14 in secondhand smoke exposure and 163 in current asthma.

†Current asthma was defined as having reported physician-diagnosed asthma and at least one of the following: asthma-like symptoms (wheeze, nocturnal chest tightness, attacks of breathlessness after activity/at rest/at night-time), asthma attacks, use of inhaled/oral medicines for breathing problems (in the last 12 months), or current use of inhalers, aerosols or tablets for asthma.

‡Individuals were categorised as being active if they exercised with a frequency of two or more times a week and with a duration of about 1 hour a week or more.

§The following illnesses were considered: hypertension, heart disease, diabetes, cancer or stroke.

¶Lung function z-scores were derived using Global Lung Initiative 2012 equations.

BMI, body mass index; FEV_1_, volume expired in the first second; FVC, forced vital capacity.

At baseline, 12% of the sample was underweight, 57% normal weight, 24% overweight and 6% obese. During follow-up almost 4% of the sample lost weight, 34% had stable weight, 53% had a moderate weight gain and 9% had a high weight gain. Table 2 shows descriptive statistics of the 16 weight change profiles identified. Almost 20% of the sample was classified in the weight change profile with baseline normal BMI and stable weight during follow-up. Out of the groups who lost weight during follow-up, obese participants at baseline were those who lost more weight over time (median −0.6 kg/year, P_25_–P_75_ −0.9 to −0.4), while among those who experienced a moderate increase in weight, median weight gain was the same in the different categories of baseline BMI. Among those with high weight gain during follow-up, overweight and obese participants at baseline were those who gained more weight. Underweight participants who lost weight or had a high weight gain represented less than 1% of the study sample and therefore were excluded from further analyses.

**Table 2:** Descriptive statistics of weight change profiles

Weight change profiles*		N (%)	Weight ECRHS I (kg) Median (P_25_; P_75_)	Weight ECRHS III (kg) Median (P_25_; P_75_)	Weight change during follow-up (kg/year) Median (P_25_; P_75_)
Underweight	Weight loss	2 (0.1)†	55.5 (54; 57)	48.5 (45; 52)	−0.3 (−0.4; −0.3)
	Stable weight	167 (4.6)	53 (50; 56)	55 (51; 59)	0.1 (0; 0.2)
	Moderate weight gain	259 (7.1)	53 (50; 58)	65.3 (60; 70.4)	0.5 (0.4; 0.7)
	High weight gain	25 (0.7)†	52 (50; 57)	78 (74; 85)	1.2 (1.1; 1.5)
Normal BMI	Weight loss	38 (1)	63.5 (60; 74)	55 (52; 65)	−0.4 (−0.4; −0.3)
	Stable weight	715 (19.5)	64 (59; 72)	65.8 (60; 74)	0.1 (0.0; 0.2)
	Moderate weight gain	1164 (31.7)	65 (60; 72)	76 (70; 84)	0.5 (0.4; 0.7)
	High weight gain	180 (4.9)	66 (60; 72)	92.4 (86; 98)	1.2 (1.1; 1.4)
Overweight	Weight loss	52 (1.4)	80 (76; 87)	71 (66; 75.8)	−0.4 (−0.6; −0.3)
	Stable weight	291 (7.9)	79 (73; 85)	80 (73; 86.8)	0.1 (−0.1; 0.2)
	Moderate weight gain	454 (12.4)	80 (73; 86)	90.9 (84; 97.1)	0.5 (0.4; 0.7)
	High weight gain	95 (2.6)	79 (70; 85)	103 (96.4; 113.9)	1.3 (1.1; 1.5)
Obese	Weight loss	46 (1.3)	95 (87; 105)	85 (72; 93)	−0.6 (−0.9; −0.4)
	Stable weight	65 (1.8)	90 (85; 100)	92 (85; 101)	0.1 (−0.1; 0.1)
	Moderate weight gain	85 (2.3)	93 (87; 103)	105 (97.1; 114)	0.5 (0.4; 0.7)
	High weight gain	35 (1)	95 (85; 109)	125 (112; 135)	1.3 (1.1; 1.8)
Overall		3673 (100)	68 (59; 78)	76 (66; 87.3)	0.4 (0.1; 0.7)

*Weight change profiles were defined combining BMI at baseline and weight change during follow-up. BMI categories at baseline: underweight: BMI <20 kg/m^2^; normal weight: 20 kg/m^2^≤BMI<25 kg/m^2^; overweight: 25 kg/m^2^ ≤BMI <30 kg/m^2^; obese: BMI ≥30 kg/m^2^. Weight change was computed as the difference between weight measured at ECRHS III and ECRHS I divided by the total duration follow-up (in years). Weight change categories: weight loss: <−0.25 kg/year; stable: within ±0.25 kg/year; moderate weight gain: 0.25–1 kg/year; high weight gain: >1 kg/year.

†Not analysed further because of small sample size.

### Associations between weight change profiles and lung function trajectories

**Figure 1:**
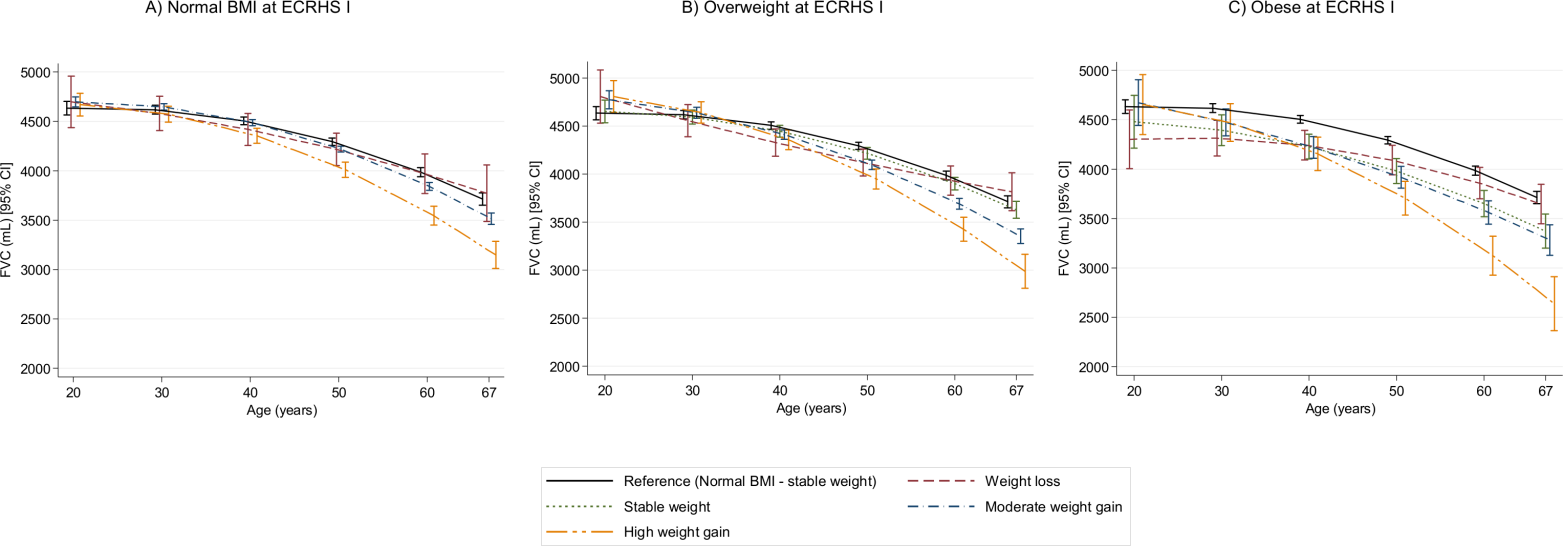
Estimated trajectories of FVC (in mL) decline by weight change profiles. The figure shows estimated FVC values and their corresponding 95% CI. Models are adjusted for sex, height, age, age squared, smoking status, an interaction term between smoking status and age, current asthma and spirometer type. Reference category: normal BMI at baseline and stable weight during follow-up. All graphs are presented with a ‘jitter’ (0.05) to avoid overlap of CI bars. BMI, body mass index; FVC, forced vital capacity.

**Figure 2:**
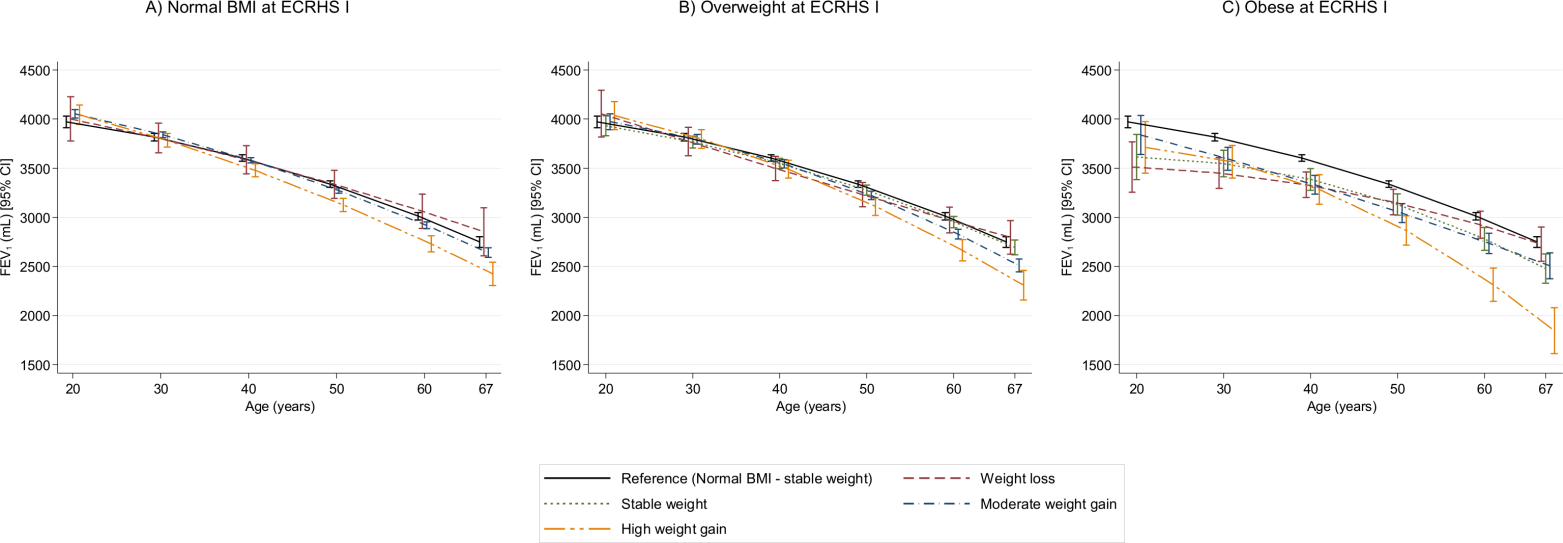
Estimated trajectories of FEV_1_ (mL) decline by weight change profiles. The figure shows estimated FEV_1_ values and their corresponding 95% CI. Models are adjusted for sex, height, age, age squared, smoking status, an interaction term between smoking status and age, current asthma and spirometer type. Reference category: normal BMI at baseline and stable weight during follow-up. All graphs are presented with a ‘jitter’ (0.05) to avoid overlap of CI bars. BMI, body mass index; FEV_1_, forced expiratory volume in 1 s.

**Figure 3:**
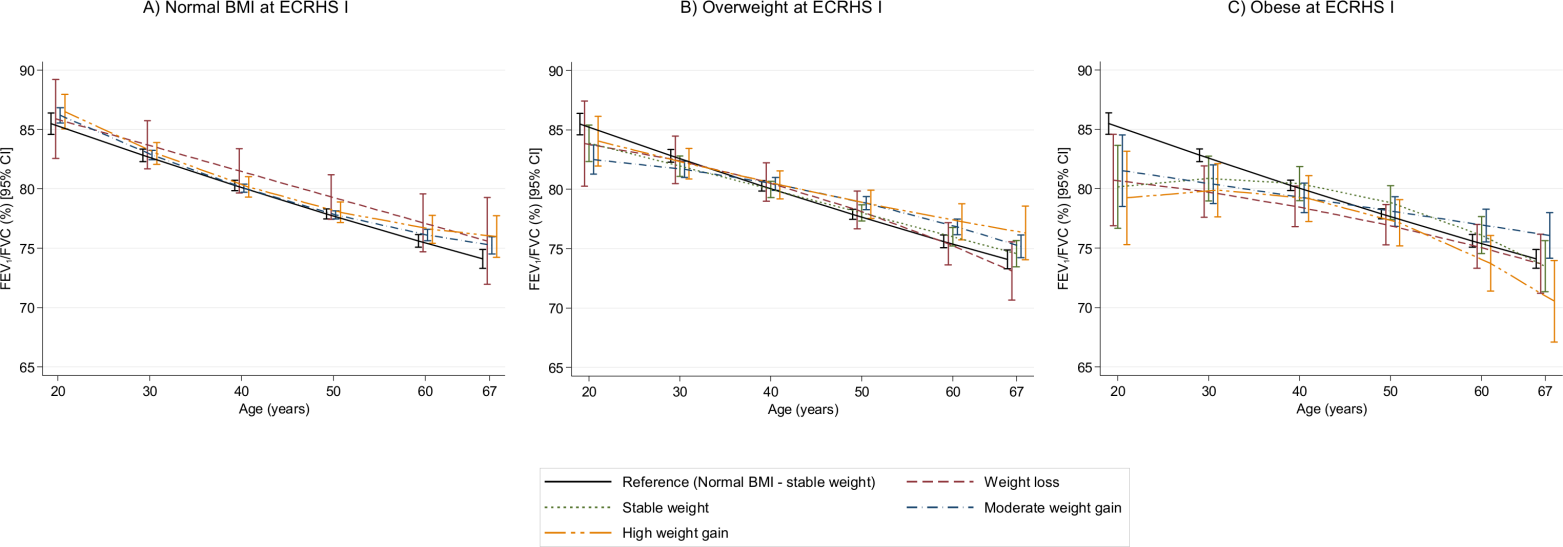
Estimated trajectories of FEV_1_/FVC (%) decline by weight change profiles. The figure shows estimated FEV_1_/FVC values and their corresponding 95% CI. Models are adjusted for sex, height, age, age squared, smoking status, an interaction term between smoking status and age, current asthma and spirometer type. Reference category: normal BMI at baseline and stable weight during follow-up. All graphs are presented with a ‘jitter’ (0.05) to avoid overlap of CI bars. BMI, body mass index; FEV_1_, forced expiratory volume in 1 s; FVC, forced vital capacity.

To facilitate interpretation of results, the estimated trajectories of lung function by weight change profiles are presented separately for normal BMI, overweight and obese categories at baseline (figures 1–3). Among adults with baseline normal BMI, overweight and obesity, those with moderate and high weight gain during follow-up exhibited significantly steeper FVC decline than those with stable weight (Panels A, B and C in figure 1). Estimated differences in FVC at 25 and 65 years by weight change profiles (see online supplementary table S3) show that, in comparison with participants with baseline normal BMI and stable weight, baseline overweight and obese participants with high weight gain had lower estimated FVC at 65 years (−677 mL (95% CI −841 to −512); p<0.001 and −1.011 mL (−1.259 to −763); p<0.001, respectively) despite similar estimated FVC levels at age 25 (see online supplementary table S3).

In contrast to weight gain, obese (but not overweight or normal BMI) adults at baseline who lost weight during follow-up exhibited an attenuation of FVC decline (panel C in figure 1). We estimated that, at age 25 years, obese participants had lower FVC levels than normal BMI participants. However, obese individuals who lost weight during follow-up were estimated to have not significantly different FVC values at age 65 years than participants with baseline normal BMI and stable weight (see online supplementary table S3).

Supplementary figure S2 shows lung function trajectories for subjects with baseline underweight. In young adulthood, participants with baseline underweight had lower estimated FVC values than baseline normal BMI participants (see online supplementary figure S2). However, baseline underweight participants with stable weight during follow-up were estimated to have very similar FVC values at age 65 to participants with baseline normal BMI and stable weight.

We found very similar results for estimated FEV_1_ trajectories (figure 2, online supplementary figure S2 and table S4). We found no evidence that FEV_1_/FVC ratio trajectories were different by weight change profiles, except for two groups. Subjects with baseline underweight who had stable weight or moderate weight gain showed a steeper decline in FEV_1_/FVC ratio than participants with baseline normal BMI and stable weight during follow-up (figure 3, online supplementary figure S2 and table S5).

Secondary analysis using lung function z-scores instead of absolute lung function showed similar results to the main analysis for all lung function parameters (see online supplementary figure S3). Stratification by sex showed that FVC and FEV_1_ decline was steeper in men who gained weight than in their female counterparts, particularly in the obese category (see online supplementary figure S4 and S5), but there was no difference with regard to the FEV_1_/FVC ratio (see online supplementary figure S6). All sensitivity analyses showed very similar results (see online supplementary figure S7–S12). However, the lung function differences between the reference category and some overweight/obese weight change profiles were attenuated when the analyses were restricted to participants who reported to be non-smokers at all examinations and when additionally adjusting for physical activity, educational level and any long-term limiting illness.

## Discussion

In this population-based study we found that weight change over a 20-year period was associated with the rate of lung function decline in adulthood. Specifically, we found that: (1) in participants with baseline normal BMI, overweight and obesity in young adulthood, moderate and high weight gain during follow-up were associated with accelerated FVC and FEV_1_ decline; (2) in participants with obesity in young adulthood, weight loss during follow-up was associated with attenuated FVC and FEV_1_ decline; (3) in underweight participants in young adulthood, stable weight during follow-up was associated with an attenuation of FVC and FEV_1_ decline; and (4) we found no evidence of an association between weight change and FEV_1_/FVC ratio decline, with the exception of underweight participants with either stable weight or moderate weight gain, both of whom exhibited accelerated FEV_1_/FVC ratio decline over follow-up.

### Interpretation

Our findings that moderate and high weight gain accelerates FVC and FEV_1_ decline and that weight loss attenuates it are consistent with previous research in young adults.^3–10^ This demonstrates how weight changes can affect lung function until late adulthood. Our approach of combining baseline BMI categories with weight change over time let us distinguish the effects of different weight change profiles on lung function throughout adult life. Two potential mechanisms have been proposed to explain the association of weight gain with accelerated lung function decline. First, weight gain can affect lung function through mechanical effects on lungs. Abdominal and thoracic fat mass are likely to reduce vital capacity by limiting the room for lung expansion during inspiration, in turn leading to expiratory flow limitation.^19^ These mechanical effects may also explain the observed sex differences in relation to lung function decline, consistent with previous studies,^4 8 20^ as men tend to accumulate more fat mass in the abdominal area than women.^21^ Second, weight gain can impair lung function by inflammatory processes, as adipose tissue is a source of inflammatory mediators^22 23^ that can damage lung tissue and reduce airway diameter.^24^ Unfortunately, we did not have measures of chest compliance or markers of systemic inflammation related to obesity, and therefore we could not disentangle the mechanical effects of body mass on lung function from the inflammatory effects.

There are some potential mechanisms that can explain the association between weight loss and attenuation of lung function decline in obese subjects. First, it is possible that weight loss reverses the mechanical effects of overweight/obesity on the respiratory system allowing the recovery of lung function. Second, weight loss may relate to a reduction of inflammatory processes in the lung which in turn can help to attenuate lung function decline related to excessive weight. This hypothesis is supported by previous research showing that lung function decline associated with air pollution, which likely affects lung function via inflammation, could be attenuated with improvement of air quality.^25^ Third, weight loss may be accompanied by improvement of metabolic alterations related to excess body weight, such as insulin dysregulation, high fasting glucose levels, hyperlipidaemia or systemic hypertension, which are also related to impaired lung function.^26 27^ Fourth, the observed association between weight loss and attenuated lung function decline could be related to confounding by changes in lifestyle (eg, increasing physical activity or changing diet) that can follow awareness of the harmful effects of overweight/obesity. Indeed, quitting smoking and becoming physically active in adulthood has been related to better lung function levels and/or attenuated lung function decline.^8 18 28 29^ Although we accounted for changes in smoking status during follow-up, levels of physical activity and presence of long-term limiting illness that could be accompanied by metabolic alterations (hypertension, heart disease, diabetes, cancer or stroke) at ECRHS II in sensitivity analyses, we did not have information on physical activity or diet at baseline. Further studies with repeated measures of lifestyle factors and indicators of metabolic dysregulation associated with weight changes are needed to disentangle the mechanisms underlying the association of weight loss and attenuated lung function decline.

We also found that stable weight during follow-up in individuals underweight in young adulthood was associated with attenuated FVC and FEV_1_ decline, while those with baseline underweight and moderate weight gain had a parallel FVC and FEV_1_ decline to individuals with baseline normal BMI in late adulthood. These findings contrast with results of a previous longitudinal study showing that increasing BMI in initially thin adults (aged 18–30) was associated with lung function improvement over 10 years.^9^ This inconsistency could be related to differences in the definition of weight gain (ie, the use of BMI gain vs weight change) and to a different baseline age range. The relationship between weight change and lung function has received little attention in healthy underweight individuals, so further research is needed to understand the effects of weight change in underweight individuals and their underlying mechanisms.

In the present analysis we did not observe statistically different FEV_1_/FVC ratio trajectories by weight change profiles, except for underweight subjects with either stable weight or moderate weight gain during follow-up, both of whom exhibited a faster FEV_1_/FVC decline over follow-up. The observed associations in underweight subjects are in line with findings of one previous study in healthy adults^9^ and allow us to hypothesise that underweight subjects could be more susceptible to the development of airflow limitation with ageing. Also, the lack of association of weight gain with the FEV_1_/FVC ratio in the present analysis is in line with previous studies showing that the FEV_1_/FVC ratio is normal in overweight and obese individuals.^19^ The lack of association of weight gain with the FEV_1_/FVC ratio could be attributed to the fact that both FVC and FEV_1_ declines were accelerated with weight gain, which could lead to a null net effect on the ratio of these two measures (as both denominator and numerator were equally affected). This pattern suggests that weight gain is likely to be related to a restrictive pattern characterised by a reduction of lung volumes with no effect on airflow limitation. This hypothesis is supported by previous evidence showing that obesity is more likely to be associated with a restrictive ventilatory pattern than an obstructive one.^30^

### Strengths and limitations

A strength of the current study is the long follow-up (up to 20 years) and the width of age distribution covering early to late adulthood. The population-based nature of the ECRHS and broad geographical representation of participants (26 centres in 12 countries in Europe and Australia) support external validity of our results. Finally, we had lung function measures at three time points, which allowed us to estimate lung function trajectories.

A limitation of this study is the use of total body weight as the main exposure. Although total body weight has been largely used in epidemiological studies as a marker of overweight and obesity, it is limited by its inability to distinguish between fat and muscle mass, which vary with age and sex^31 32^ and could have different effects on lung function, as previously shown in children.^33^ Also, we defined weight change categories using only weight measures at baseline and last follow-up to capture ‘stable’ weight change patterns and facilitate the interpretability of our results. Of note, the correlation between individual weight change per year (taking into account three weight measurements) and the weight change variable used in our analysis was 0.998, which justifies our approach. However, we recognise that our approach precludes us from determining how long it takes for a change in weight to affect lung function decline. Given the lack of standard references for weight change in adults, we categorised weight change based on a previous longitudinal study,^17^ limiting the interpretation of our findings to our definition of ‘stable weight’ (±0.25 kg/year). However, the results were very similar when repeating our analysis using a wider category for ‘stable weight’ (±0.50 kg/year), suggesting that our findings are robust even with a less restrictive definition of ‘stable weight’. Our results may also be affected by selection bias, as participants were more likely to be highly educated and less likely to be current smokers or to be exposed to secondhand smoke than those lost to follow-up. Because these factors have been previously associated with lung function, our associations could be underestimates of the true associations in the general population. Although we accounted for a wide range of confounders, our results could be affected by potential residual confounding by, for example, dietary intake, which affects both body weight and lung function, as the available data on diet were limited to a small subset of the study sample at ECRHS II and III. Moreover, the spirometers used for lung function assessment were changed in some centres, which could have led to systematic differences inherent in lung function measurement that may differ by age and height.^34^ However, when we adjusted our analysis for spirometer type and when we replicated the analyses using lung function values corrected for change in spirometer we obtained consistent results. Finally, we used three repeated measures of lung function from a sample aged 20–44 years (mean (SD) age 34.3 (7.1) years) at baseline and 39–67 years (mean (SD) age 54.3 (7.1) years) at the last follow-up to estimate lung function trajectories throughout adulthood. However, few participants were aged around 20 years at baseline and around 67 years at the last follow-up, and in consequence the models had fewer observations at the age ends than between 30 and 60 years, where most of the observations were.

## Conclusion

In conclusion, this prospective population-based study shows that moderate and high weight gain over a 20-year period was associated with accelerated lung function decline in adulthood, while weight loss was related to its attenuation. Our findings, together with the existing literature, reinforce the public health message that overweight and obesity have deleterious effects on health, including respiratory health. However, the negative effects of overweight and obesity on lung function can be reversed by weight loss even later in adult life. Therefore, public health policies that promote healthy lifestyles and body weight may be important for maintaining good lung function in adult life.
