# Supplementary material for: Body mass index and weight change are associated with adult lung function trajectories: the prospective ECRHS study
Source: Thorax. 2020 Feb 25;75(4):313–20. doi: 10.1136/thoraxjnl-2019-213880 (PMC7231449; doi:10.1136/thoraxjnl-2019-213880)
Supplement: Supplementary data [file thoraxjnl-2019-213880supp001.pdf]

Body mass index and weight change are associated with adult lung function trajectories: the prospective ECRHS study

Gabriela P. Peralta, Alessandro Marcon, Anne-Elie Carsin, Michael J Abramson, Simone Accordini, André FS Amaral, Josep M. Antó, Gayan Bowatte, Peter Burney, Angelo G. Corsico, Pascal Demoly, Shyamali Dharmage, Bertil Forsberg, Elaine Fuertes, Vanessa Garcia-Larsen, Thorarinn Gíslason, José-Antonio Gullón, Joachim Heinrich, Mathias Holm, Deborah L. Jarvis, Christer Janson, Rain Jogi, Ane Johannessen, Bénédicte Leynaert, Jesús Martínez-Moratalla, Dennis Nowak, Nicole Probst-Hensch, Chantal Raherison-Semjen, José-Luis Sánchez-Ramos, Torben Sigsgaard, Valérie Siroux, Giulia Squillacioti, Isabel Urrutia, Joost Weyler, Jan-Paul Zock, Judith Garcia-Aymerich

ONLINE SUPPLEMENT

Contents

Methods: sensitivity analyses

Figure S1. **Flowchart of the study sample**

Table S1. **Instruments used at spirometry examinations in the ECRHS**

Table S2. **Baseline characteristics of participants included and excluded of the analysis**

Figure S2. **Estimated trajectories of FVC (first panel), FEV_1 (second panel) and FEV_1/FVC (third panel) decline in baseline underweight participants with stable weight and moderate weight gain during follow-up**

Table S3. **Estimated FVC (mL) differences among weight change profiles at age 25 years and 65 years**

Table S4. **Estimated FEV_1 (mL) differences among weight change profiles at age 25 years and 65 years**

Table S5. **Estimated FEV_1/FVC (%) differences among weight change profiles at age 25 years and 65 years**

Figure S3. **Estimated trajectories of FVC (first panel), FEV_1 (second panel) and FEV_1/FVC (third panel) decline, by weight change profiles – Using lung function standard deviation score (z-score) as outcome variable**

Figure S4. **Estimated trajectories of FVC (mL) decline, by weight change profiles - Stratified by sex**

Figure S5. **Estimated trajectories of FEV_1 (mL) decline, by weight change profiles - Stratified by sex**

Figure S6. **Estimated trajectories of FEV_1/FVC (%) decline, by weight change profiles - Stratified by sex**

Figure S7. Estimated trajectories of FVC (first panel), FEV_1 (second panel) and FEV_1/FVC (third panel) decline, by weight change profiles - Excluding participants with current asthma at any visit (n= 709)

Figure S8. Estimated trajectories of FVC (first panel), FEV_1 (second panel) and FEV_1/FVC (third panel) decline, by weight change profiles - Excluding the symptomatic arm of ECRHS (n=536)

Figure S9. Estimated trajectories of FVC (first panel), FEV_1 (second panel) and FEV_1/FVC (third panel) decline, by weight change profiles – Restricting models to participants who reported to be non-smokers at all visits (n=1,491)

Figure S10. Estimated trajectories of FVC (first panel), FEV_1 (second panel) and FEV_1/FVC (third panel) decline, by weight change profiles – Models additionally adjusted for educational level at ECRHS I and physical activity and any long-term limiting illness (hypertension/heart disease/diabetes/cancer/stroke) at ECRHS II (n=1,525)

Figure S11. Estimated trajectories of FVC (first panel), FEV_1 (second panel) and FEV_1/FVC (third panel) decline, by weight change profiles – Using lung function values corrected for change in spirometer

Figure S12. Estimated trajectories of FVC (first panel), FEV_1 (second panel) and FEV_1/FVC (third panel) decline, by weight change profiles – Using alternative categories

for weight change (weight loss: <-0.5 kg/year; stable weight ±0.5 kg/year; moderate weight gain: 0.5 to 1 kg/year; high weight gain: >1kg/year)

Local Principal Investigators, senior scientific teams and funding agencies for the European Community Respiratory Health Survey (ECRHS

Methods: sensitivity analyses

To assess the robustness of our results, we performed several sensitivity analyses. First, we excluded subjects with asthma and subjects from the symptomatic arm of the ECRHS in separate analyses to assess whether results were sensitive to the exclusion of these subsamples. Second, we restricted the final models to participants who reported being non-smokers at the three examinations to account for potential residual confounding by smoking and weight change related to change in smoking status. Third, we additionally adjusted models for educational level, physical activity and presence of any long-term limiting illness to rule out potential residual confounding. These variables were not included in the main models because they reduced the statistical power without substantially altering the results. Fourth, to account for potential misclassification in lung function due to change in spirometers over time we replicated our models using lung function values corrected for change in spirometer. These corrected values were derived using a similar methodology as previously described for another similar adult cohort.[1] Finally, we repeated our analysis defining 'stable weight' as change over time ±0.50kg/year [2] to account for potential misclassification in weight change categories (i.e., using a less restrictive definition of change 'stable weight').

References:

1 Bridevaux P-O, Dupuis-Lozeron E, Schindler C, *et al.* Spirometer Replacement and Serial Lung Function Measurements in Population Studies: Results From the SAPALDIA Study. *Am J Epidemiol* 2015;**181**:752–61. doi:10.1093/aje/kwu352

2 Nanri A, Mizoue T, Takahashi Y, *et al.* Weight change and all-cause, cancer and cardiovascular disease mortality in Japanese men and women: The Japan Public Health Center-Based Prospective Study. *Int J Obes* 2010;**34**:348–56. doi:10.1038/ijo.2009.234

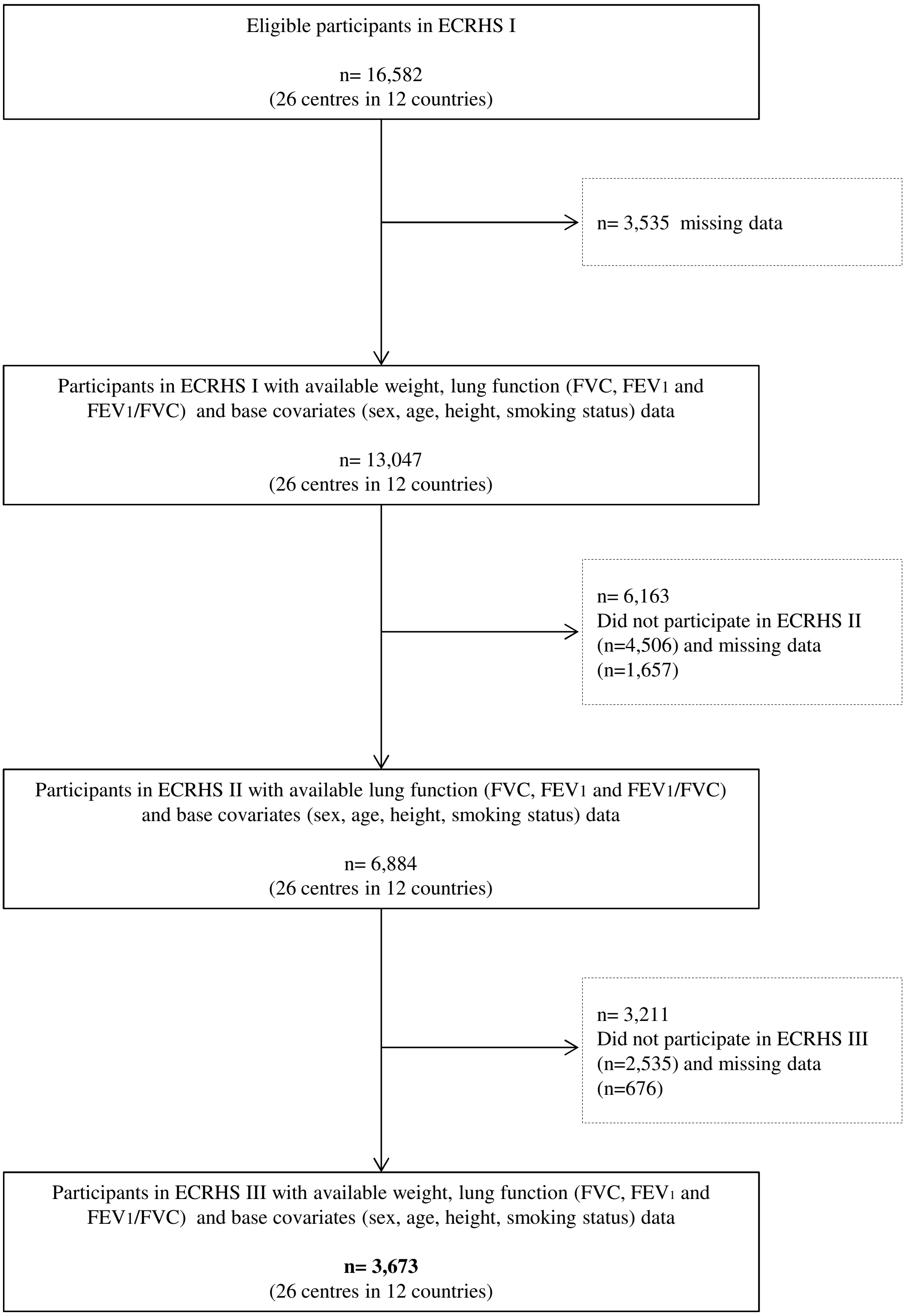

Figure 1. Flowchart of the study sample

Table S1. Instruments used at spirometry examinations in the ECRHS

Study centre	Instrument used at ECRHS I	Instrument used at ECRHS II	Instrument used at ECRHS III
Albacete	Biomedin spiro	Biomedin spiro	NDD
Anterwep City	SensorMedics displacement	Jaeger pneum	NDD
Anterwep South	SensorMedics displacement	Jaeger pneum	NDD
Barcelona	Biomedin spiro	Biomedin spiro	NDD
Basel	SensorMedics hot wire	SensorMedics hot wire	NDD
Bergen	SensorMedics displacement	SensorMedics displacement	NDD
Bordeaux	Vitalograph spiro	Vitalograph spiro	NDD
Erfurt	Jaeger pneum	Jaeger pneum	NDD
Galdakao	Biomedin spiro	Biomedin spiro	NDD
Gothenburg	SensorMedics displacement	SensorMedics displacement	NDD
Grenoble	Biomedin spiro	Biomedin spiro	NDD
Hamburg	Jaeger pneum	Jaeger pneum	NDD
Huelva	Biomedin spiro	Biomedin spiro	NDD
Ipswich	Biomedin spiro	Biomedin spiro	NDD
Melbourne	Fleisch pneumotach	SensorMedics displacement	NDD
Montpellier	Biomedin spiro	Biomedin spiro	NDD
Norwich	Biomedin spiro	Biomedin spiro	NDD
Oviedo	Biomedin spiro	Biomedin spiro	NDD
Paris	Biomedin spiro	Biomedin spiro	NDD
Pavia	Biomedin spiro	Biomedin spiro	NDD
Reykjavik	SensorMedics displacement	SensorMedics displacement	NDD
Tartu	Jaeger pneum	Jaeger pneum	NDD
Turin	Biomedin spiro	Biomedin spiro	Biomedin spiro
Umea	SensorMedics displacement	SensorMedics displacement	NDD
Uppsala	SensorMedics displacement	SensorMedics displacement	NDD
Verona	Biomedin spiro	Biomedin spiro	Biomedin spiro

Table S2. Baseline (ECRHS I) characteristics of participants included and excluded of the analysis

Characteristics	Included (n=3,673) n (%) or mean (SD)	Excluded (n=12,909) n (%) or mean (SD)	p-value
Symptomatic study arm	544 (14.8)	1,842 (14.3)	0.409
Sex. Women	1,956 (53.3)	6,6694 (51.9)	0.134
Age in years	34.3 (7.1)	33.4 (7.2)	<0.001
Height in cm	170.6 (9.4)	170.7 (9.7)	0.557
Weight in kg	69.5 (13.5)	69.5 (13.9)	0.842
BMI			
Continuous, in kg/m2	23.8 (3.7)	23.8 (3.9)	0.864
Underweight	453 (12.3)	1,412 (13.3)	0.512
Normal weight	2,097 (57.1)	5,987 (56.2)	
Overweight	892 (24.3)	2,562 (24.1)	
Obese	231 (6.3)	684 (6.4)	
Smoking status			
Non-smoker	1,651 (45.0)	5,199 (40.3)	<0.001
Ex-smoker	818 (22.3)	2,545 (19.7)	
Current smoker	1,204 (32.8)	5,149 (39.9)	
Second-hand smoke exposure. Yes	1,939 (52.9)	7,526 (58.6)	<0.001
Current asthma*. Yes	378 (10.5)	1,329 (10.6)	0.880
Age completed full time education			
<17 years	675 (21.5)	2,644 (24.3)	<0.001
17-20 years	1,205 (38.4)	4,514 (41.5)	
>20 years	1,256 (40.1)	3,709 (34.1)	
Lung function			
FVC (ml)	4,516 (988)	4,517 (1,038)	0.957
FEV_1 (ml)	3,702 (798)	3,716 (845)	0.360
FEV_1/FVC (%)	82.3 (6.9)	82.5 (7.5)	0.080

*Current asthma was defined as having reported physician-diagnosed asthma and at least one of the following: asthma-like symptoms (wheeze, nocturnal chest tightness, attacks of breathlessness after activity/at rest/at night-time), asthma attacks, use of inhaled/oral medicines for breathing problems (in the last 12 months), or current use of inhalers, aerosols or tablets for asthma
Abbreviations: BMI, body mass index; FEV1, volume expired in the first second; FVC, forced vital capacity; SD, standard deviation

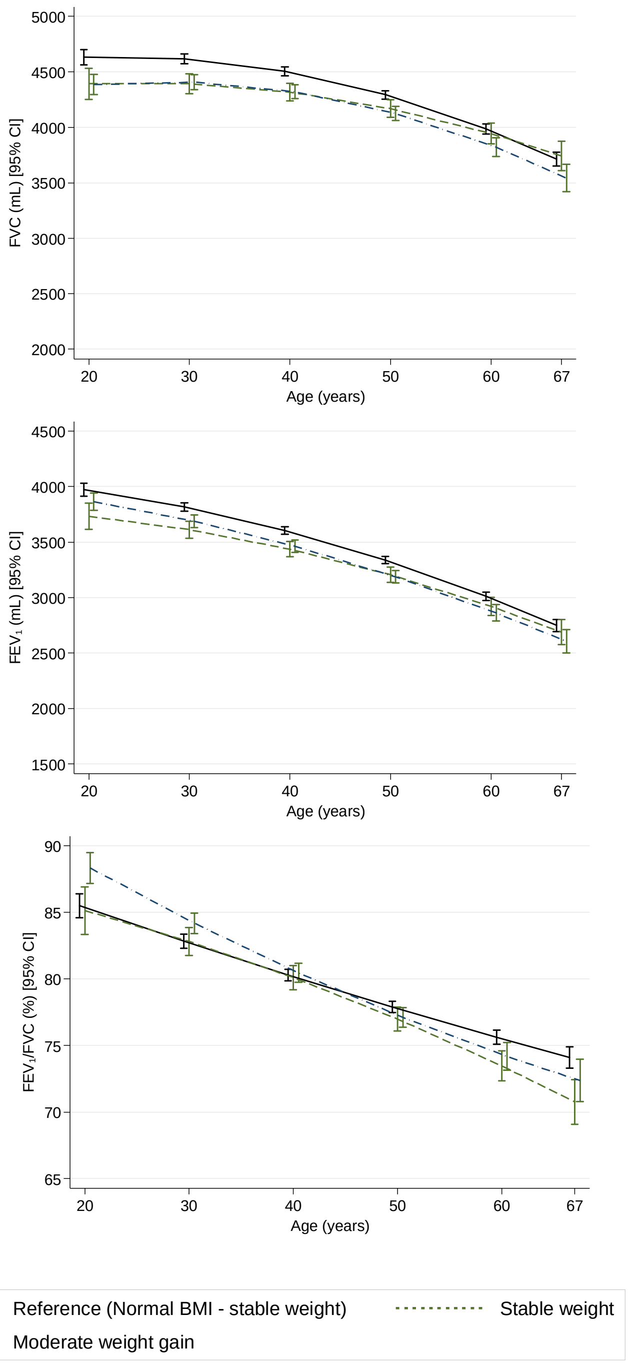

Figure S2. Estimated trajectories of FVC (first panel), FEV_1 (second panel) and FEV_1/FVC (third panel) decline in baseline underweight participants with stable weight and moderate weight gain during follow-up
Models are adjusted for the same variables than main models (see Figures 1 to 3).

Table S3. Estimated FVC (mL) differences among weight change profiles at age 25 years and 65 years

		25 years		65 years	
**Weight change profiles**		**Coef (95% CI)**	**p-value**	**Coef (95% CI)**	**p-value**
Normal BMI	Stable weight	Reference		Reference	
Underweight †	Stable weight	-236 [-354 to -118]	<0.001	9 [-117 to 134]	0.891
	Moderate weight gain	-228 [-318 to -138]	<0.001	-167 [-285 to -50]	0.005
Normal BMI	Weight loss	6 [-204 to 216]	0.957	36 [-223 to 294]	0.788
	Moderate weight gain	47 [-17 to 112]	0.150	-182 [-249 to -115]	<0.001
	High weight gain	-2 [-106 to 102]	0.971	-528 [-658 to -398]	<0.001
Overweight	Weight loss	40 [-176 to 256]	0.716	53 [-132 to 238]	0.574
	Stable weight	-5 [-107 to 98]	0.930	-84 [-176 to 8]	0.073
	Moderate weight	79 [-9 to 166]	0.077	-342 [-423 to -260]	<0.001
	High weight gain	100 [-39 to 239]	0.158	-677 [-841 to -512]	<0.001
Obese	Weight loss	-320 [-552 to -87]	0.007	-84 [-274 to 107]	0.389
	Stable weight	-189 [-396 to 18]	0.074	-338 [-502 to -174]	<0.001
	Moderate weight	-58 [-238 to 122]	0.529	-429 [-576 to -282]	<0.001
	High weight gain	-58 [-296 to 180]	0.632	-1,011 [-1,259 to -763]	<0.001

Coefficients represent the estimated differences of FVC (mL) for each one of the weight change profiles compared to individuals with baseline normal BMI and stable weight during follow-up. Models are adjusted for sex, height, age, age squared, smoking status, an interaction term between smoking status and age, current asthma and spirometer type.

† Underweight who lost weight and underweight with high weight gain were excluded from multivariate analyses because of small sample size.

Abbreviations: FVC, forced vital capacity; 95% CI, 95% confidence interval

Table S4. Estimated FEV_1 (mL) differences among weight change profiles at age 25 years and 65 years

		25 years		65 years	
**Weight change profiles**		**Coef (95% CI)**	**p-value**	**Coef (95% CI)**	**p-value**
Normal BMI	Stable weight	Reference		Reference	
Underweight †	Stable weight	-222 [-324 to -120]	<0.001	-70 [-178 to 39]	0.208
	Moderate weight gain	-119 [-197 to -41]	0.003	-146 [-247 to -45]	0.005
Normal BMI	Weight loss	7 [-175 to 189]	0.940	87 [-136 to 309]	0.445
	Moderate weight gain	53 [-3 to 108]	0.064	-105 [-163 to -47]	<0.001
	High weight gain	19 [-72 to 109]	0.688	-313 [-424 to -201]	<0.001
Overweight	Weight loss	12 [-174 to 199]	0.899	19 [-141 to 179]	0.817
	Stable weight	-44 [-133 to 44]	0.327	-57 [-136 to 22]	0.159
	Moderate weight	-7 [-82 to 69]	0.861	-222 [-293 to -152]	<0.001
	High weight gain	25 [-96 to 145]	0.687	-413 [-554 to -271]	<0.001
Obese	Weight loss	-412 [-612 to -211]	<0.001	-41 [-205 to 124]	0.628
	Stable weight	-308 [-487 to -130]	0.001	-257 [-399 to -115]	<0.001
	Moderate weight	-181 [-337 to -26]	0.022	-254 [-381 to -127]	<0.001
	High weight gain	-245 [-451 to -40]	0.019	-839 [-1,053 to -626]	<0.001

Coefficients represent the estimated differences of FEV_1 (mL) for each one of the weight change profiles compared to individuals with baseline normal BMI and stable weight during follow-up. Models are adjusted for sex, height, age, age squared, smoking status, an interaction term between smoking status and age, current asthma and spirometer type.

† Underweight who lost weight and underweight with high weight gain were excluded from multivariate analyses because of small sample size.

Abbreviations: FEV_1, volume expired in the first second; 95% CI, 95% confidence interval

Table S5. Estimated FEV_1/FVC (%) differences among weight change profiles at age 25 years and 65 years

		25 years		65 years	
**Weight change profiles**		**Coef (95% CI)**	**p-value**	**Coef (95% CI)**	**p-value**
Normal BMI	Stable weight	Reference		Reference	
Underweight†	Stable weight	-0.1 [-1.6 to 1.3]	0.872	-3 [-4.5 to -1.4]	0.000
	Moderate weight gain	2.1 [1 to 3.2]	0.000	-1.7 [-3.2 to -0.2]	0.028
Normal BMI	Weight loss	0.7 [-1.9 to 3.2]	0.617	1.5 [-1.8 to 4.8]	0.364
	Moderate weight gain	0.3 [-0.5 to 1.1]	0.440	0.9 [0.1 to 1.8]	0.028
	High weight gain	0.5 [-0.8 to 1.8]	0.432	1.6 [0.0 to 3.2]	0.055
Overweight	Weight loss	-0.9 [-3.6 to 1.8]	0.509	-0.7 [-3 to 1.5]	0.527
	Stable weight	-1.2 [-2.5 to 0.1]	0.063	0.5 [-0.7 to 1.6]	0.425
	Moderate weight	-2 [-3.1 to -0.9]	0.000	1.1 [0.1 to 2.2]	0.026
	High weight gain	-1.1 [-2.8 to 0.6]	0.221	2.1 [0.0 to 4.1]	0.051
Obese	Weight loss	-3.9 [-6.8 to -0.9]	0.010	-0.4 [-2.7 to 1.9]	0.726
	Stable weight	-3.5 [-6.1 to -0.9]	0.009	-0.2 [-2.2 to 1.7]	0.807
	Moderate weight	-3.2 [-5.5 to -0.9]	0.006	1.8 [0.0 to 3.6]	0.053
	High weight gain	-4.4 [-7.4 to -1.5]	0.003	-3 [-6.1 to 0.1]	0.055

Coefficients represent the estimated differences of FEV_1/FVC (%) for each one of the weight change profiles compared to individuals with baseline normal BMI and stable weight during follow-up. Models are adjusted for sex, height, age, age squared, smoking status, an interaction term between smoking status and age, current asthma and spirometer type.

† Underweight who lost weight and underweight with high weight gain were excluded from multivariate analyses because of small sample size.

Abbreviations: FEV_1, volume expired in the first second; FVC, forced vital capacity; 95% CI, 95% confidence interval

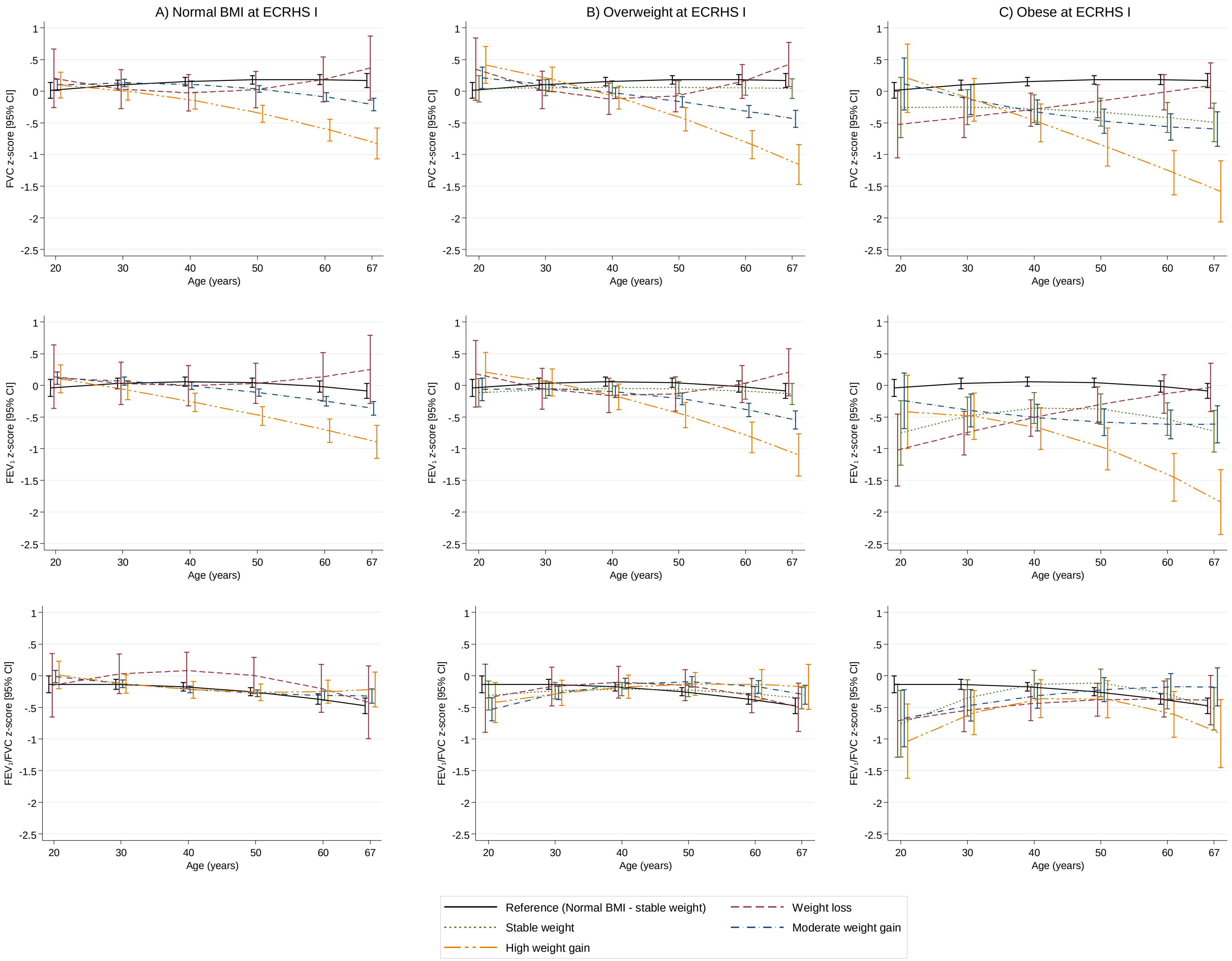

Figure S3. Estimated trajectories of FVC (first panel), FEV_1 (second panel) and FEV_1/FVC (third panel) decline, by weight change profiles– Using lung function standard deviation score (z-score) as outcome variable. Models are adjusted for the same variables as in the main models, except sex and height (see Figures 1 to 3).

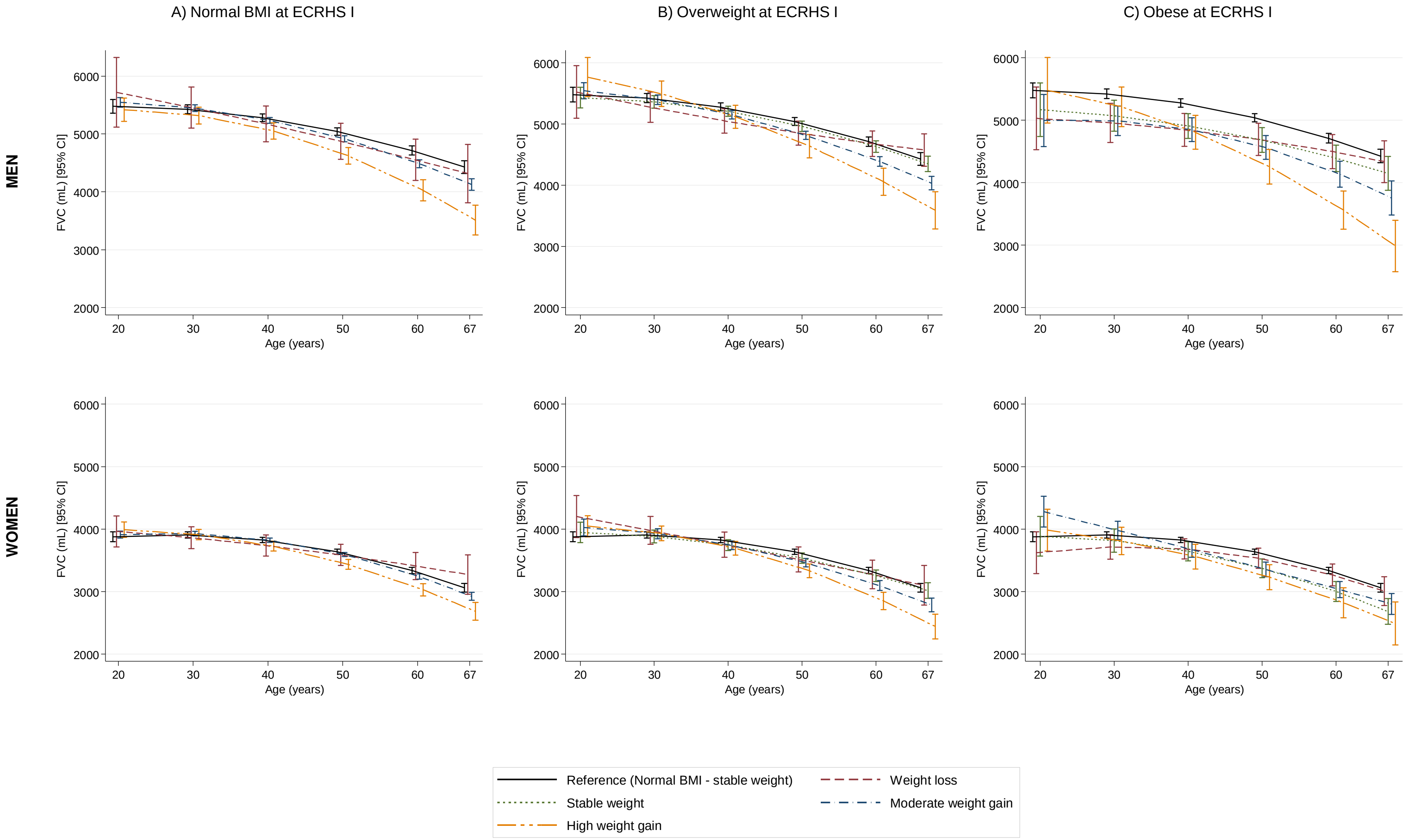

Figure S4. Estimated trajectories of FVC (mL) decline, by weight change profiles – Stratified by sex
Models are adjusted for the same variables as in the main models, except sex (see Figure 1). P-value for sex interaction: 0.124

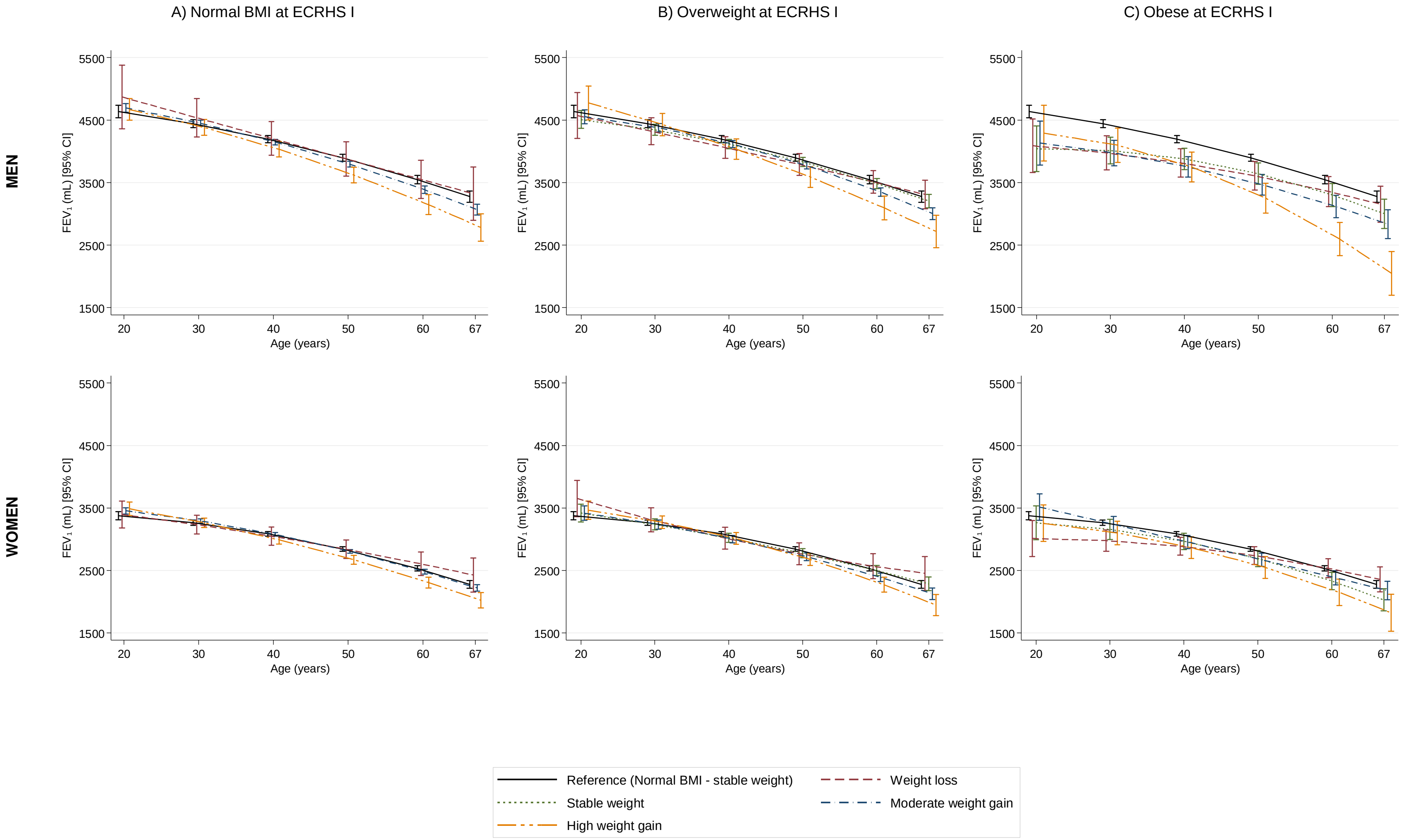

Figure S5. Estimated trajectories of FEV1 (mL) decline, by weight change profiles– Stratified by sex
Models are adjusted for the same variables as in the main models, except sex (see Figure 2). P-value for sex interaction: 0.006

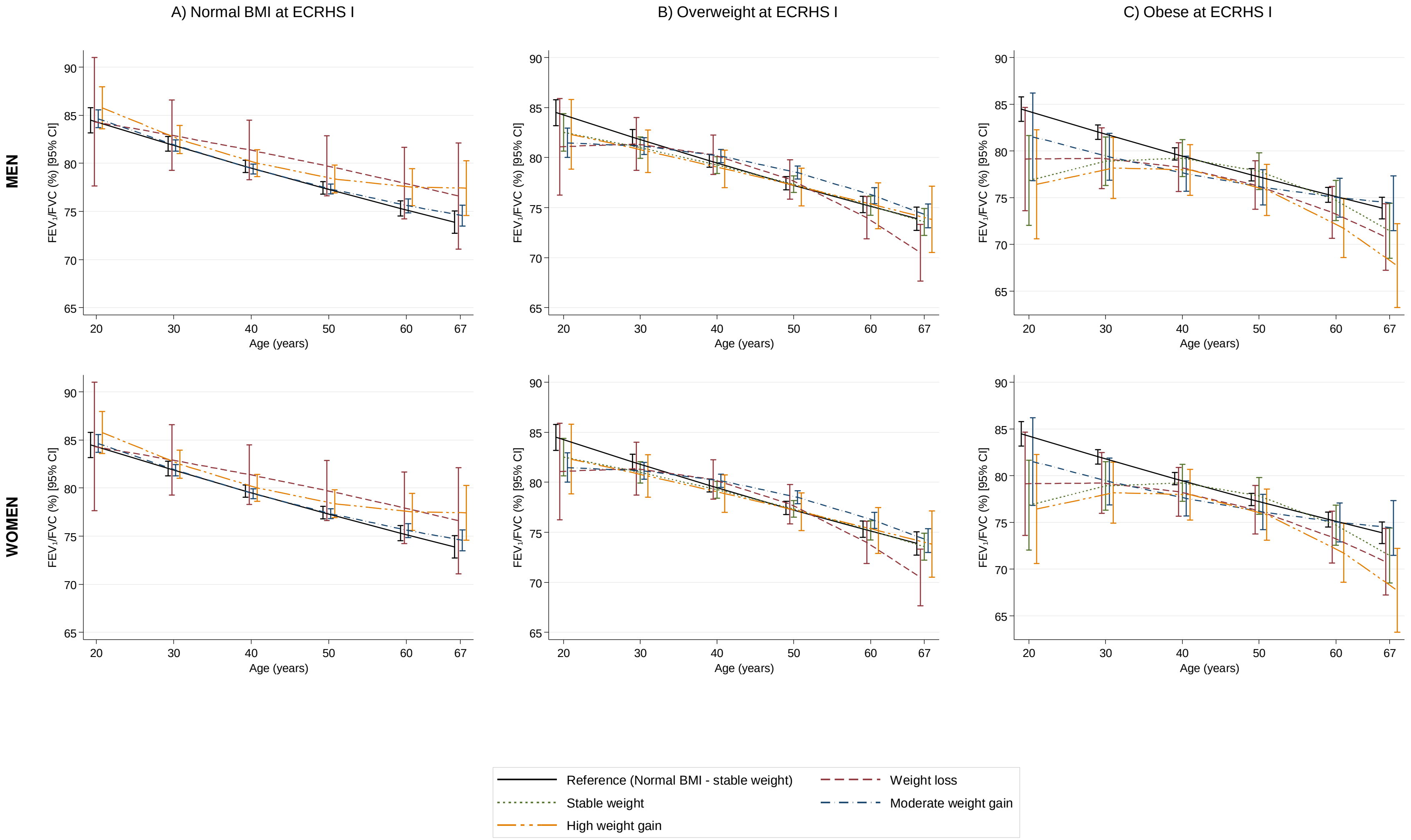

Figure S6. Estimated trajectories of FEV1/FVC (%) decline, by weight change profiles– Stratified by sex.
Models are adjusted for the same variables as in the main models, except sex (see Figure 3). P-value for sex interaction: 0.247

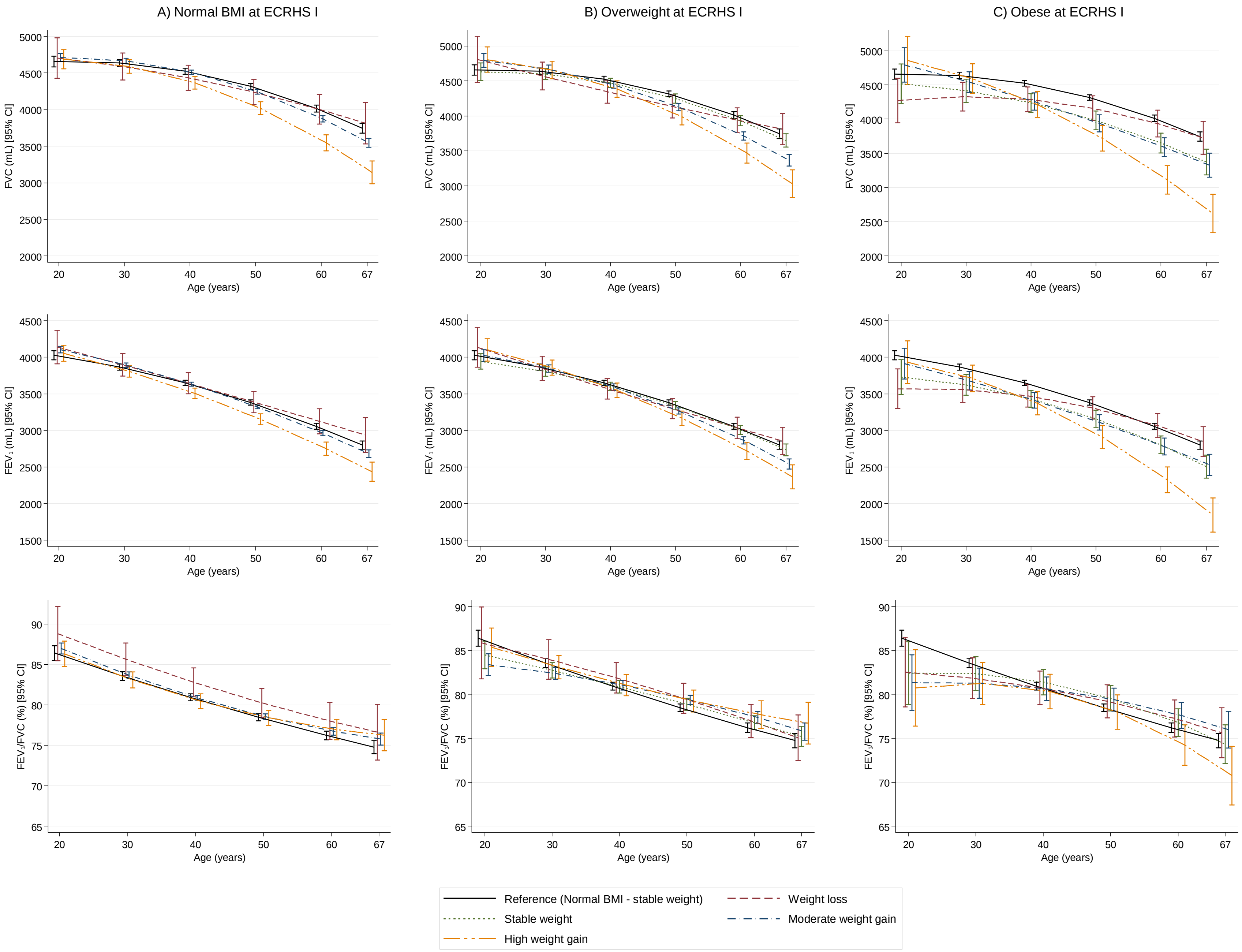

Figure S7. Estimated trajectories of FVC (first panel), FEV_1 (second panel) and FEV_1/FVC (third panel) decline, by weight change profiles - Excluding participants with current asthma at any visit (n= 709). Models are adjusted for the same variables as in the main models (see Figures 1 to 3).

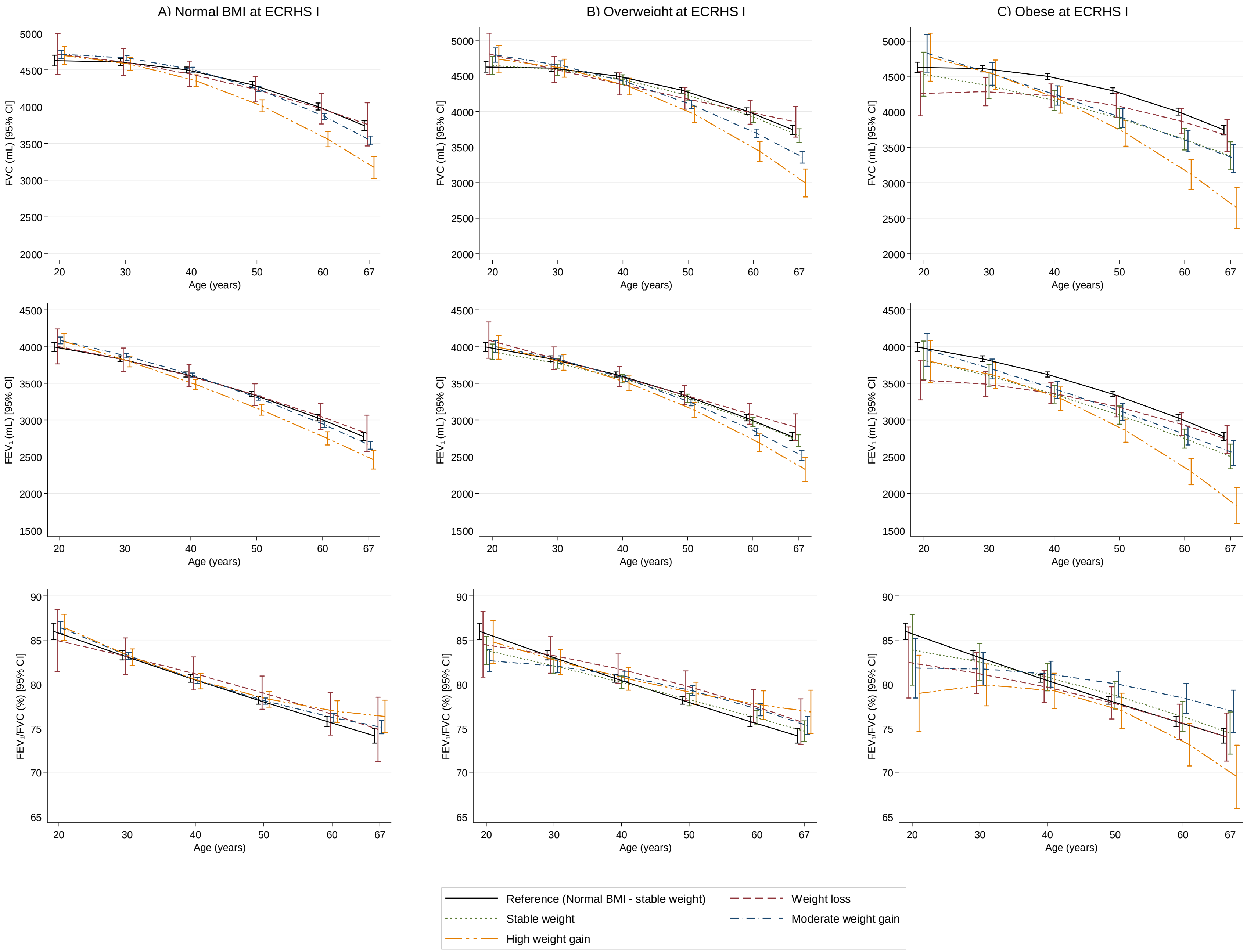

Figure S8. Estimated trajectories of FVC (first panel), FEV_1 (second panel) and FEV_1/FVC (third panel) decline, by weight change profiles - Excluding the symptomatic arm of ECRHS (n=536). Models are adjusted for the same variables as in the main models (see Figures 1 to 3).

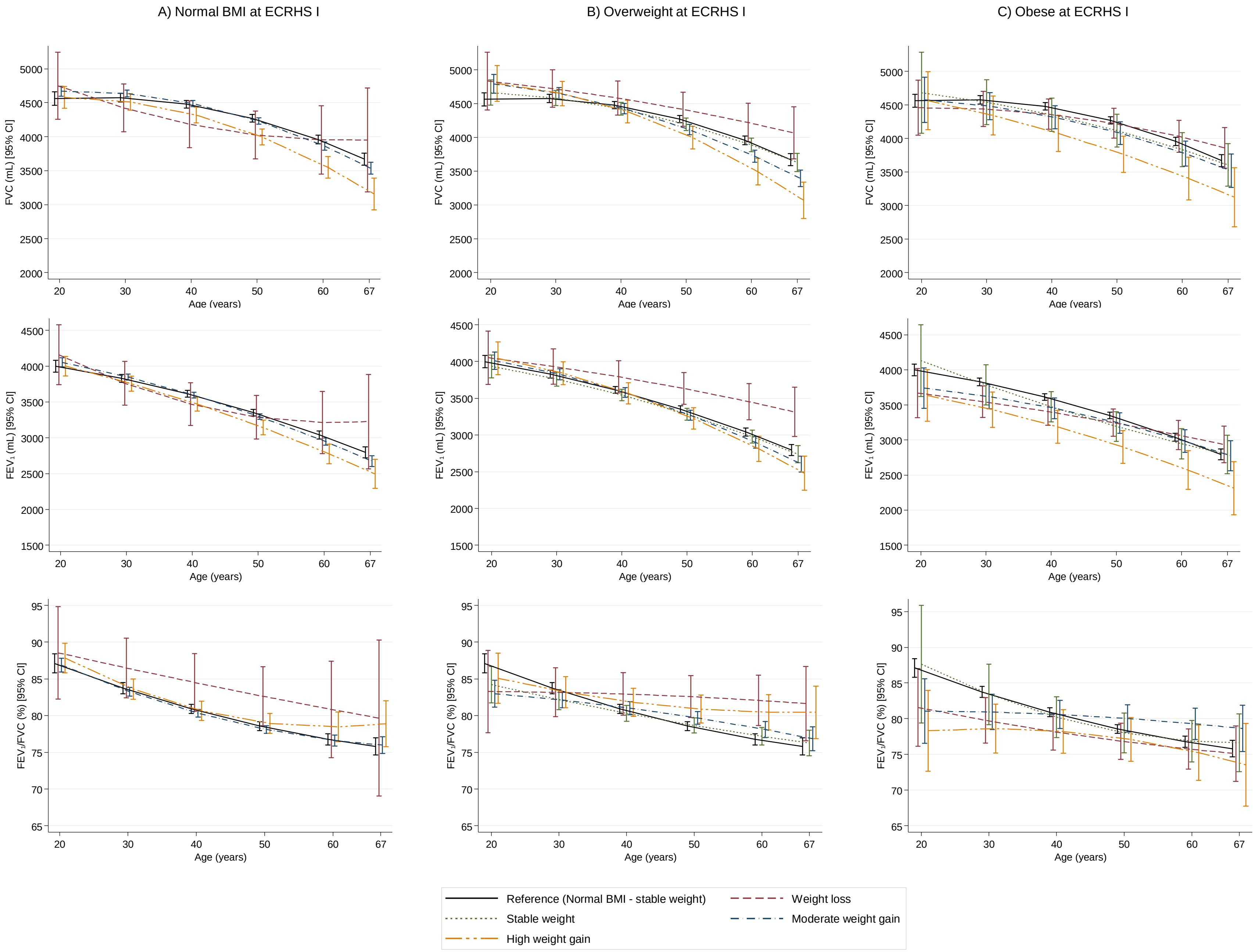

Figure S9. Estimated trajectories of FVC (first panel), FEV_1 (second panel) and FEV_1/FVC (third panel) decline, by weight change profiles – Restricting models to participants who reported to be non-smokers at all visits (n=1,491). Models are adjusted for the same variables as in the main models, except smoking status (see Figures 1 to 3).

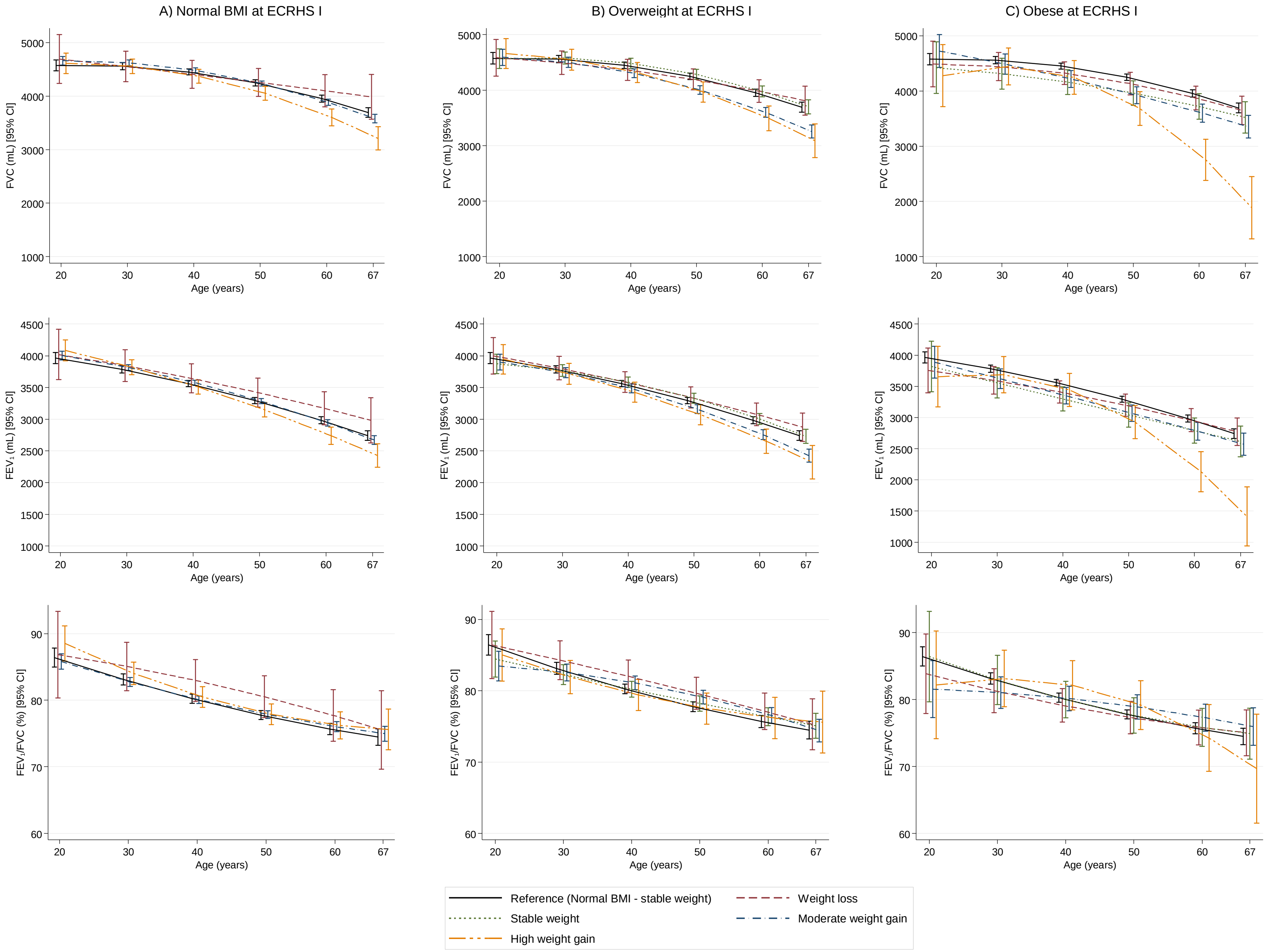

Figure S10. Estimated trajectories of FVC (first panel), FEV_1 (second panel) and FEV_1/FVC (third panel) decline, by weight change profiles – Models additionally adjusted for educational level at ECRHS I and physical activity and any long-term limiting illness (hypertension/heart disease/diabetes/cancer/stroke) at ECRHS II (n=1,525). Models are adjusted for the same variables as in the main models (see Figures 1 to 3).

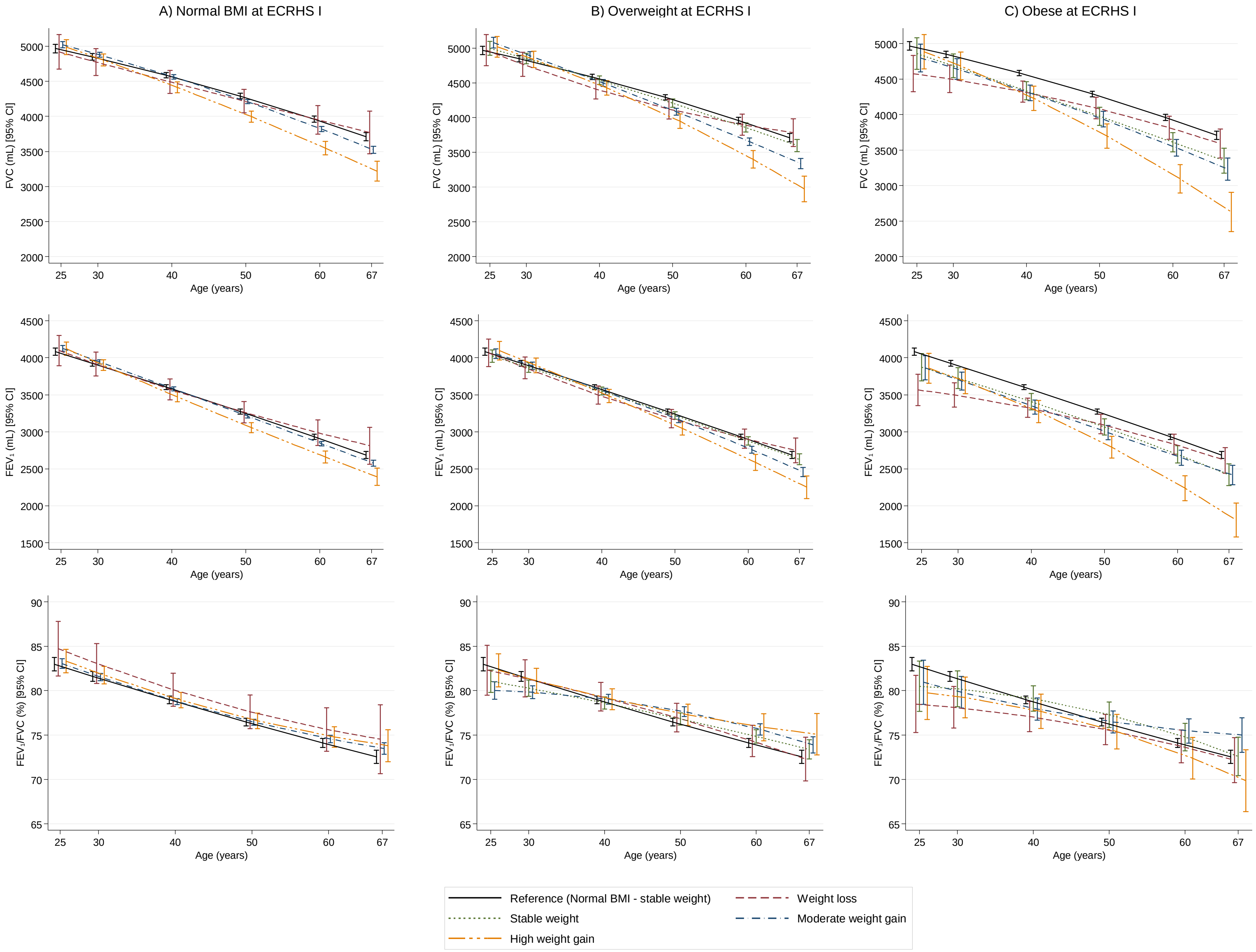

Figure S11. Estimated trajectories of FVC (first panel), FEV_1 (second panel) and FEV_1/FVC (third panel) decline, by weight change profiles – Using lung function values corrected for change in spirometer. Models are adjusted for the same variables as in the main models, except for spirometer type (see Figures 1 to 3). Lung function trajectories start at age 25 years because corrected values were calculated only for subjects aged ≥25 year at baseline.

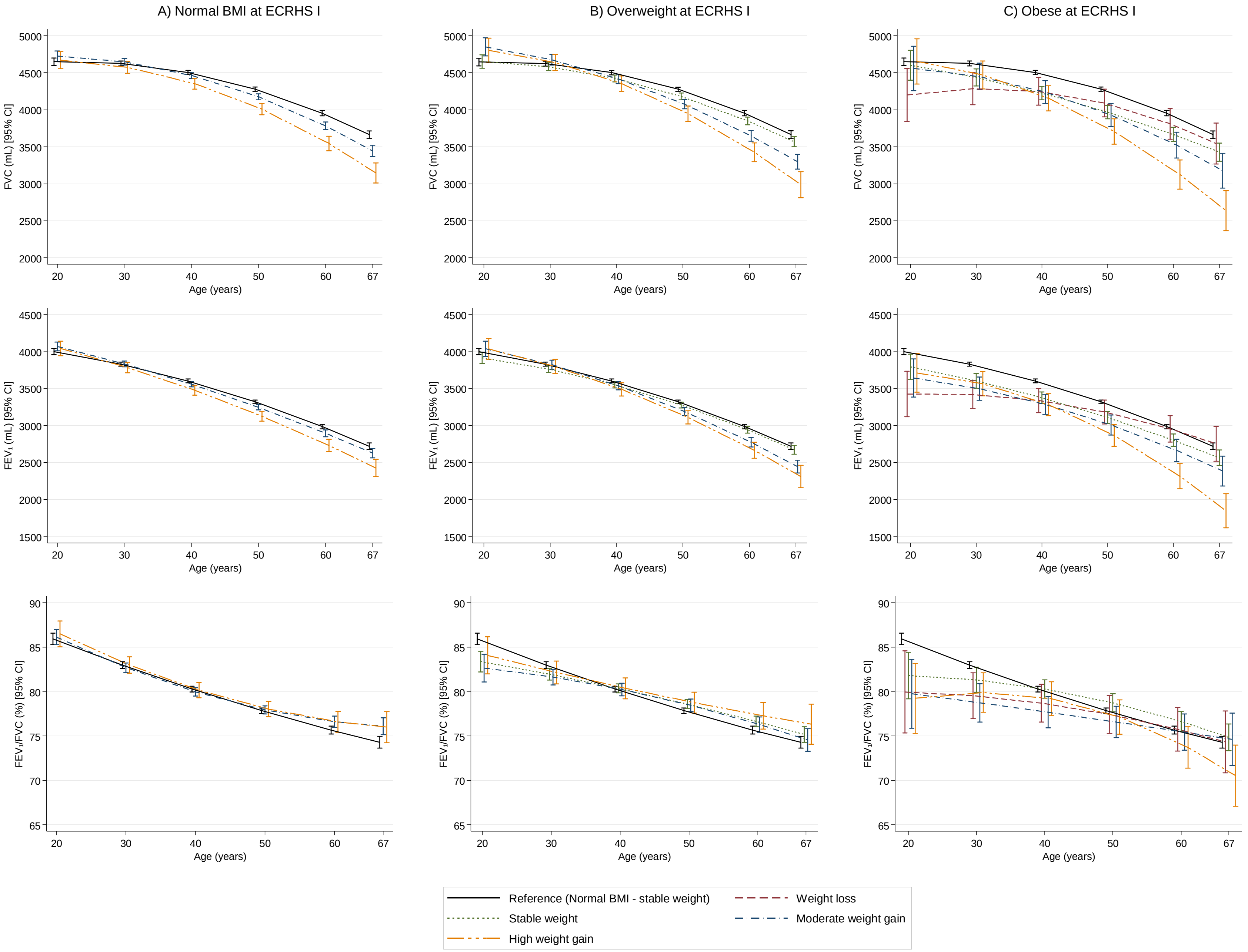

Figure S12. Estimated trajectories of FVC (first panel), FEV_1 (second panel) and FEV_1/FVC (third panel) decline, by weight change profiles – Using alternative categories for weight change (weight loss: <-0.5 kg/year; stable weight ±0.5 kg/year; moderate weight gain: 0.5 to 1 kg/year; high weight gain: >1kg/year). Models are adjusted for the same variables as in the main models (see Figures 1 to 3). Normal BMI and overweight subjects who lost weight were excluded due to small sample size.

Local Principal Investigators, senior scientific teams and funding agencies for the European Community Respiratory Health Survey (ECRHS)

ECRHS I

Co-ordinating Centre (London): P Burney, S Chinn, C Luczynska†, D Jarvis, E Lai.

Project Management Group: P Burney (Project leader-UK), S Chinn (UK), C Luczynska † (UK), D Jarvis (UK), P Vermeire† (Antwerp), H Kesteloot (Leuven), J Bousquet (Montpellier), D Nowak (Hamburg), J Prichard † (Dublin), R de Marco† (Verona), B Rijcken (Groningen), JM Anto (Barcelona), J Alves (Oporto), G Boman (Uppsala), N Nielsen (Copenhagen), P Paoletti (Pisa).

Financial support: The following grants helped to fund the local studies. **Australia**: Asthma Foundation of Victoria, Allen and Hanbury's, **Belgium**: Belgian Science Policy Office, National Fund for Scientific Research, **Estonia:** Estonian Science Foundation, grant no 1088, **France:** Ministère de la Santé, Glaxo France, Insitut Pneumologique d'Aquitaine, Contrat de Plan Etat-Région Languedoc-Rousillon, CNMATS, CNMRT (90MR/10, 91AF/6), Ministre delegué de la santé, RNSP, France; GSF, **Germany**: Bundesminister für Forschung und Technologie, **Italy:** Ministero dell'Università e della Ricerca Scientifica e Tecnologica, CNR, Regione Veneto grant RSF n. 381/05.93, **Norway:** Norwegian Research Council project no. 101422/310, **Spain:** Fondo de Investigación Sanitaria (#91/0016-060-05/E, 92/0319 and #93/0393), Hospital General de Albacete, Hospital General Juan Ramón Jiménez, Dirección Regional de Salud Pública (Consejería de Sanidad del Principado de Asturias), CIRIT (1997 SGR 00079) and Servicio Andaluz de Salud, **Sweden:** The Swedish Medical Research Council, the Swedish Heart Lung Foundation, the Swedish Association against Asthma and Allergy, **Switzerland:** Swiss national Science Foundation grant 4026-28099, **UK:** National Asthma Campaign, British Lung Foundation, Department of Health, South Thames Regional Health Authority.

Coordination: The co-ordination of this work was supported by the European Commission and the authors and participants are grateful to the late C. Baya and M. Hallen for their help during the study and K. Vuylsteek and the members of the COMAC for their support.

ECRHS II

Steering Committee: U. Ackermann-Liebrich (University of Basel, Switzerland); N. Kuenzli (University of Basel, and University of Southern California, Los Angeles, USA); J.M. Antó and J. Sunyer (Institut Municipal d' Investigació Médica (IMIM-IMAS), Universitat Pompeu Fabra (UPF), Spain); P. Burney (project leader), S Chinn, D. Jarvis, J. Knox and C. Luczynska (King's College London, UK); I. Cerveri (University of Pavia, Italy); R. de Marco† (University of Verona, Italy); T. Gislason (Iceland University Hospital, Iceland); J. Heinrich and M. Wjst (GSF–Institute of Epidemiology, Germany); C. Janson (Uppsala University, Sweden); B. Leynaert and F. Neukirch (Institut National de la Sante´ et de la Recherche Meidicale (INSERM),France); J. Schouten (University of Groningen, The Netherlands); C. Svanes (University of Bergen, Norway); P. Vermeire† (University of Antwerp, Belgium).

Principal Investigators and senior scientific teams: **Australia:** (M. Abramson, E.H Walters, J. Raven); **Belgium: South Antwerp and Antwerp City** (P. Vermeire, J. Weyler, M. van Sprundel, V. Nelen); **Estonia: Tartu** (R. Jõgi, A. Soon); **France: Paris** (F. Neukirch, B. Leynaert, R. Liard, M. Zureik), **Grenoble** (I. Pin, J. Ferran-Quentin), **Bordeaux** (A. Taytard,

C.Raherison), **Montpellier** (J.Bousquet, PJ Bousquet); **Germany: Erfurt** (J. Heinrich, M. Wjst, C. Frye, I. Meyer); **Iceland: Reykjavik** (T. Gislason, E. Bjornsson, D. Gislason, K.B **Jörundsdóttir***); ***Italy: Turin** (R. Bono, M. Bugiani, P.Piccioni, E. Caria, A. Carosso, E. Migliore, G. Castiglioni), **Verona** (R. de Marco†, G. Verlato, E. Zanolin, S. Accordini, A. Poli, V. Lo Cascio, M. Ferrari, I. Cazzoletti), **Pavia** (A. Marinoni, S. Villani, M. Ponzio, F. Frigerio, M. Comelli, M. Grassi, I. Cerveri, A. Corsico); **Norway: Bergen** (A. Gulsvik, E. Omenaas, C. Svanes, B. Laerum); **Spain: Albacete** (J. Martinez-Moratalla Rovira, E. Almar, M. Arévalo, C. Boix, G González, J.M. Ignacio García, J. Solera, J Damián), **Galdakao** (N. Muñozguren, J. Ramos, I. Urrutia, U. Aguirre), **Barcelona** (J. M. Antó, J. Sunyer, M. Kogevinas, J. P. Zock, X. Basagaña, A. Jaen, F. Burgos, C. Acosta), **Huelva** (J. Maldonado, A. Pereira, J.L. Sanchez), **Oviedo** (F. Payo, I . Huerta, A. de la Vega, L Palenciano, J Azofra, A Cañada); **Sweden**: **Göteborg** (K. Toren,L. Lillienberg, A. C. Olin, B. Balder, A. Pfeifer-Nilsson, R. Sundberg), **Umea** (E. Norrman, M. Soderberg, K.A Franklin, B. Lundback, B. Forsberg, L. Nystrom), **Uppsala** (C. Janson, G. Boman, D. Norback, G. Wieslander, M. Gunnbjornsdottir); **Switzerland: Basel** (N. Küenzli, B. Dibbert, M. Hazenkamp, M. Brutsche, U. Ackermann-Liebrich); **United Kingdom: Ipswich** (D. Jarvis, R. Hall, D. Seaton), **Norwich** (D. Jarvis, B. Harrison).

Financial Support: **Australia:** National Health and Medical Research Council; **Belgium:** Antwerp: Fund for Scientific Research (grant code, G.0402.00), University of Antwerp, Flemish Health Ministry; **Estonia: Tartu** Estonian Science Foundation grant no 4350; **France: (All)** Programme Hospitalier de Recherche Clinique—Direction de la Recherche Clinique (DRC) de Grenoble 2000 number 2610, Ministry of Health, Ministère de l'Emploi et de la Solidarité, Direction Génerale de la Santé, Centre Hospitalier Universitaire (CHU) de Grenoble, **Bordeaux**: Institut Pneumologique d'Aquitaine, **Grenoble:** Comite des Maladies Respiratoires de l'Isere, **Montpellier:** Aventis (France), Direction Regionale des Affaires Sanitaires et Sociales Languedoc-Roussillon**, Paris:** Union Chimique Belge- Pharma (France), Aventis (France), Glaxo France; **Germany: Erfurt** GSF—National Research Centre for Environment and Health, Deutsche Forschungsgemeinschaft (grant code, FR1526/1-1), **Hamburg:** GSF—National Research Centre for Environment and Health, Deutsche Forschungsgemeinschaft (grant code, MA 711/4-1); **Iceland: Reykjavik**, Icelandic Research Council, Icelandic University Hospital Fund; **Italy: Pavia** GlaxoSmithKline Italy, Italian Ministry of University and Scientific and Technological Research (MURST),Local University Funding for Research 1998 and 1999, **Turin**: Azienda Sanitaria Locale 4 Regione Piemonte (Italy), Azienda Ospedaliera Centro Traumatologico Ospedaliero/Centro Traumatologico Ortopedico—Istituto Clinico Ortopedico Regina Maria Adelaide Regione Piemonte, **Verona:** Ministero dell'Universita´ e della Ricerca Scientifica (MURST), Glaxo Wellcome spa; **Norway: Bergen:** Norwegian Research Council, Norwegian Asthma and Allergy Association, Glaxo Wellcome AS, Norway Research Fund; **Spain:** Fondo de Investigacion Santarias (grant codes, 97/0035-01,99/0034-01 and 99/0034 02), HospitalUniversitario de Albacete, Consejeria de Sanidad, **Barcelona:** Sociedad Espanola de Neumologı´a y Cirugı´a Toracica, Public Health Service(grant code, R01 HL62633-01), Fondo de Investigaciones Santarias (grant codes, 97/0035-01, 99/0034-01, and 99/0034-02), Consell Interdepartamentalde Recerca i Innovacio´ Tecnolo`gica (grant code, 1999SGR 00241) Instituto de Salud Carlos III; Red deCentros de Epidemiologı´a y Salud Pu´blica, C03/09,Redde Basesmoleculares y fisiolo´gicas de lasEnfermedadesRespiratorias,C03/011and Red de Grupos Infancia y Medio Ambiente G03/176, **Huelva:** Fondo de Investigaciones Santarias (grant codes, 97/0035-01, 99/0034-01, and 99/0034-02), **Galdakao:** Basque Health Department, **Oviedo:** Fondo de Investigaciones Sanitaria (97/0035-02, 97/0035, 99/0034-01, 99/0034-02, 99/0034-04, 99/0034-06, 99/350, 99/0034--07), European Commission (EU-PEAL PL01237), Generalitat de Catalunya (CIRIT 1999 SGR 00214), Hospital Universitario de Albacete, Sociedad Española de Neumología y Cirugía Torácica (SEPAR R01 HL62633-01) Red de Centros de Epidemiología y Salud Pública (C03/09), Red de Bases moleculares y fisiológicas de las Enfermedades Respiratorias (C03/011) and Red de Grupos Infancia y Medio Ambiente (G03/176);97/0035-01, 99/0034-01,

and99/0034-02); **Sweden: Göteborg, Umea, Uppsala**: Swedish Heart Lung Foundation, Swedish Foundation for Health Care Sciences and Allergy Research, Swedish Asthma and Allergy Foundation, Swedish Cancer and Allergy Foundation, Swedish Council for Working Life and Social Research (FAS); **Switzerland: Basel** Swiss National Science Foundation, Swiss Federal Office for Education and Science, Swiss National Accident Insurance Fund; **UK: Ipswich and Norwich**: Asthma UK (formerly known as National Asthma Campaign).

Coordination: The coordination of this work was supported by the European Commission, as part of their Quality of Life programme, (Grant code: QLK4-CT-1999-01237).

ECRHS III

Principal Investigators and senior scientific teams: **Australia: Melbourne** (M.Abramson, G. Benke, S. Dharmage, B. Thompson, S. Kaushik, M Matheson. **Belgium: South Antwerp & Antwerp City** (J. Weyler, H.Bentouhami, V. Nelen). **Estonia:** Tartu (R. Jõgi, H.Orru). **France: Bordeaux** (C. Raherison, P.O Girodet) **Grenoble** (I. Pin, V. Siroux, J.Ferran, J.L Cracowski) **Montpellier** (P. Demoly,A.Bourdin, I. Vachier) **Paris** (B. Leynaert, D. Soussan, D. Courbon, C. Neukirch, L. Alavoine, X. Duval, I. Poirier). **Germany: Erfurt** (J .Heinrich, E. Becker, G. Woelke, O. Manuwald) **Hamburg** (H. Magnussen, D. Nowak, A-M Kirsten). **Iceland: Reykjavik** (T. Gislason, B. Benediktsdottir, D. Gislason, E.S Arnardottir, M. Clausen, G. Gudmundsson, L. Gudmundsdottir, H. Palsdottir, K. Olafsdottir, S. Sigmundsdottir, K. Bara-Jörundsdottir). **Italy**: **Pavia** (I .Cerveri, A.Corsico, A. Grosso, F. Albicini, E. Gini, E.M Di Vincenzo, V. Ronzoni, S. Villani, F. Campanella, M. Gnesi, F. Manzoni, L. Rossi, O. Ferraro) **Turin:** (M. Bugiani, R. Bono, P. Piccioni, R. Tassinari, V. Bellisario, G. Trucco) **Verona:** (R de Marco†, S. Accordini, L. Calciano, L. Cazzoletti, M. Ferrari, A.M Fratta Pasini, F. Locatelli, P. Marchetti, A. Marcon, E. Montoli, G. Nguyen, M. Olivieri, C. Papadopoulou, C.Posenato, G. Pesce, P. Vallerio, G. Verlato, E. Zanolin). **Norway:** (C. Svanes, E. Omenaas, A. Johannessen, T. Skorge, F. Gomez Real). **Spain: Albacete** (J. Martinez-Moratalla Rovira, E. Almar, A. Mateos, S. García, A. Núñez, P.López, R. Sánchez, E Mancebo), **Barcelona:** (J-M. Antó, J.P Zock, J Garcia-Aymerich, M Kogevinas, X. Basagaña, A.E. Carsin, F. Burgos, C. Sanjuas, S Guerra, B. Jacquemin, P.Davdand, **Galdakao**: N. Muñozguren, I. Urrutia, U. Aguirre, S. Pascual), **Huelva**:(J Antonio Maldonado, A. Pereira, J. Luis Sánchez, L. Palacios, **Oviedo**: (F. Payo, I. Huerta, N. Sánchez, M. Fernández, B. Robles). **Sweden: Göteborg** (K. Torén, M. Holm, J-L Kim, A-C. Olin, A. Dahlman-Höglund), **Umea** (B. Forsberg, L. Braback, L Modig, B Järvholm, H Bertilsson, K.A Franklin, C Wahlgreen), **Uppsala:** (B Andersson, D Norback, U Spetz Nystrom, G Wieslander, G.M Bodinaa Lund, K Nisser); **Switzerland: Basel** (N.M. Probst-Hensch, N. Künzli, D. Stolz, C. Schindler, T. Rochat, J.M. Gaspoz, E. Zemp Stutz, M.Adam, C. Autenrieth, I. Curjuric, J. Dratva, A. Di Pasquale, R. Ducret-Stich, E. Fischer, L. Grize, A. Hensel, D. Keidel, A. Kumar, M. Imboden, N. Maire, A. Mehta, H. Phuleria, M. Ragettli, M. Ritter, E. Schaffner, G.A Thun, A. Ineichen, T. Schikowski, M. Tarantino, M. Tsai. **UK: London** (P. Burney, D. Jarvis, S. Kapur, R. Newson, J. Potts), **Ipswich:** (N. Innes), **Norwich**: (A. Wilson).

Financial Support: **Australia**: National Health & Medical Research Council. **Belgium: Antwerp South, Antwerp City:** Research Foundation Flanders (FWO), grant code G.0.410.08.N.10 (both sites). **Estonia: Tartu-** SF0180060s09 from the Estonian Ministry of Education. **France:** (**All**) Ministère de la Santé. Programme Hospitalier de Recherche Clinique (PHRC) national 2010. **Bordeaux:** INSERM U897 Université Bordeaux segalen, **Grenoble:** Comite Scientifique AGIRadom 2011. **Paris:** Agence Nationale de la Santé, Région Ile de France, domaine d'intérêt majeur (DIM). **Germany: Erfurt:** German Research Foundation HE 3294/10-1 **Hamburg**: German Research Foundation MA 711/6-1, NO 262/7-1. **Iceland:**

Reykjavik, The Landspitali University Hospital Research Fund, University of Iceland Research Fund, ResMed Foundation, California, USA, Orkuveita Reykjavikur (Geothermal plant), Vegagerðin (The Icelandic Road Administration (ICERA). **Italy:** All Italian centres were funded by the Italian Ministry of Health, Chiesi Farmaceutici SpA, in addition **Verona** was funded by Cariverona foundation, Education Ministry (MIUR). **Norway:** Norwegian Research council grant no 214123, Western Norway Regional Health Authorities grant no 911631, Bergen Medical Research Foundation. **Spain:** Fondo de Investigación Sanitaria (PS09/02457, PS09/00716 09/01511) PS09/02185 PS09/03190), Servicio Andaluz de Salud , Sociedad Española de Neumología y Cirurgía Torácica (SEPAR 1001/2010). Fondo de Investigación Sanitaria (PS09/02457), **Barcelona:** Fondo de Investigación Sanitaria (FIS PS09/00716), **Galdakao:** Fondo de Investigación Sanitaria (FIS 09/01511) **Huelva**: Fondo de Investigación Sanitaria (FIS PS09/02185) and Servicio Andaluz de Salud **Oviedo:** Fondo de Investigación Sanitaria (FIS PS09/03190). **Sweden:** All centres were funded by The Swedish Heart and Lung Foundation, The Swedish Asthma and Allergy Association, The Swedish Association against Lung and Heart Disease, Swedish Research Council for health, working life and welfare (FORTE) **Göteborg :** Also received further funding from the Swedish Council for Working life and Social Research. Umea also received funding from Vasterbotten Country Council ALF grant. **Switzerland:** The Swiss National Science Foundation (grants no 33CSCO-134276/1, 33CSCO-108796, 3247BO-104283, 3247BO-104288, 3247BO-104284, 3247-065896, 3100-059302, 3200-052720, 3200-042532, 4026-028099) The Federal office for forest, environment and landscape, The Federal Office of Public Health, The Federal Office of Roads and Transport, the canton’s government of Aargan, Basel-Stadt, Basel-Land, Geneva, Luzern, Ticino, Valais and Zürich, the Swiss Lung League, the canton's Lung League of Basel Stadt/ Basel, Landschaft, Geneva, Ticino, Valais and Zurich, SUVA, Freiwillige Akademische Gesellschaft, UBS Wealth Foundation, Talecris Biotherapeutics GmbH, Abbott Diagnostics, European Commission 018996 (GABRIEL), Wellcome Trust WT 084703MA. **UK:** Medical Research Council (Grant Number 92091). Support also provided by the National Institute for Health Research through the Primary Care Research Network.

Coordination: The coordination was funded through the Medical Research Council (Grant Number 92091).

† Deceased
